# Using Unbiased Chemical Proteomics Approaches to Explore the Target Landscape of the Resistance Refractory 7‑Azaindole MMV022224 in *Plasmodium falciparum*

**DOI:** 10.1021/acsinfecdis.6c00276

**Published:** 2026-06-25

**Authors:** Aisha J. Syed, Rachel Milne, Victoriano Corpas-Lopez, Gourav Dey, Nonlawat Boonyalai, Richard J. Wall, Natalie Wiedemar, Lukas Montejo, Flore Nardella, Kathryn J. Wicht, Charisse Flerida A. Pasaje, John Okombo, Jacquin C. Niles, David A. Fidock, Marcus C. S. Lee, Debopam Chakrabarti, Stephen Patterson, Susan Wyllie

**Affiliations:** a Drug Discovery Unit, Division of Drug Discovery, Faculty of Life Sciences, 3042 University of Dundee, Dow Street, Dundee DD1 5EH, United Kingdom; b Division of Biological Chemistry and Drug Discovery, Faculty of Life Sciences, 3042 University of Dundee, Dow Street, Dundee DD1 5EH, United Kingdom; c Division of Molecular Microbiology, Burnett School of Biomedical Sciences, 6243 University of Central Florida, 12722 Research Parkway, Orlando, Florida 32826, United States; d Holistic Drug Discovery and Development (H3D) Centre, Department of Chemistry and Institute of Infectious Disease and Molecular Medicine, 37716 University of Cape Town, Rondebosch 7701, South Africa; e Department of Biological Engineering, 2167 Massachusetts Institute of Technology, Cambridge, Massachusetts 02139, United States; f Department of Microbiology & Immunology, Columbia University Irving Medical Center, New York, New York 10032, United States; g Center for Malaria Therapeutics and Antimicrobial Resistance, Division of Infectious Diseases, Department of Medicine, Columbia University Irving Medical Center, New York, New York 10032, United States

**Keywords:** chemical pulldown, Plasmodium, chemical biology, isothermal TPP, target deconvolution, antimalarial drug discovery

## Abstract

Current standard of care, artemisinin-based therapies for malaria, are threatened by emerging drug resistance. Developing antimalarials with novel mechanisms of action and low propensity for resistance is of the highest priority. Here, we explore the target landscape of MMV022224, a promising antimalarial that is active against multiple stages of *Plasmodium falciparum* and refractory to resistance generation. Using two orthogonal chemical proteomics approaches, chemical pulldown and thermal proteome profiling, we demonstrate that MMV022224 binds selectively and with high affinity to the genetically essential *P. falciparum* protein kinase 6 (*Pf*PK6), as well as to several additional *Plasmodium* kinases. Enzymatic studies verify that MMV022224 inhibits *Pf*PK6; however, *Pf*PK6 knockdown does not affect parasite compound susceptibility, confirming that *Pf*PK6 inhibition is not the sole driver of antimalarial activity and that MMV022224 may act through broader, kinase-focused polypharmacology. Employing the same chemical proteomics strategies, we demonstrate that the structurally related azaindole, TCMDC-135051, is a selective inhibitor of the cyclin-dependent kinase *Pf*CLK3. Collectively, these studies demonstrate the value of chemical proteomics for antimalarial drug target deconvolution.

## Graphical abstract

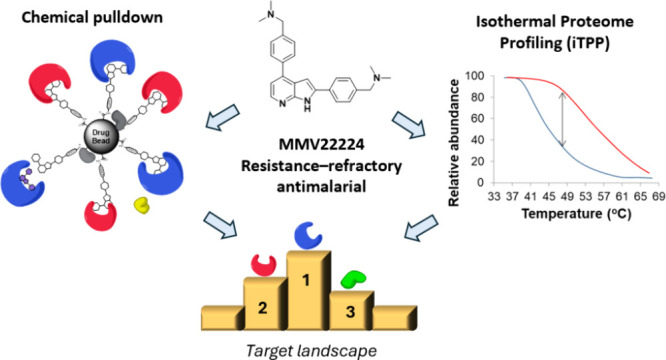

## Introduction

Malaria remains the world’s largest parasitic killer. In 2025, the World Health Organization reported 282 million cases and ∼610,000 deaths.^1^ Children under the age of five and pregnant women are the most vulnerable to this disease which results from infection with protozoan parasites of the *Plasmodium* genus. The majority of malaria deaths are caused by *P. falciparum* . While the past decade has seen significant progress in reducing the global burden of malaria, progress has stalled and even reversed due to a number of events including the SARS-CoV-2 pandemic and severe flooding.^2^ Perhaps most concerning of all is the emergence of resistance to the current standard of care artemisinin-based combination therapies (ACT). Clinical artemisinin resistance is now prevalent in Southeast Asia^3^ and mutations within the gene encoding *P. falciparum* Kelch 13 associated with resistance are increasingly prevalent in some parts of eastern and southern Africa.^4–6^ Developing new, effective antimalarials that can treat artemisinin-refractory infections is of the highest priority. In addition, there is a pressing need for medicines that provide chemoprotection, prevent transmission, and treat *P. vivax* -mediated relapses.

Identifying new chemical start points for antimalarial drug discovery is principally achieved through phenotypic screening of large, chemically diverse compound libraries.^7^ This approach has been necessitated by the relative dearth of robustly validated drug targets in *Plasmodium* thus limiting opportunities for target-focused strategies. To leverage promising hits that emerge from phenotypic screening and to expand the pool of chemically validated targets, drug target deconvolution studies are often initiated. The frontline approach for drug target deconvolution in *Plasmodium* is via *in vitro* evolution of resistance followed by whole-genome sequencing analysis (IVIEWGA).^8^ In general, large cultures (10^8^–10^9^) of genetically diverse (nonclonal) parasites are exposed to lethal concentrations of a phenotypically active compound until a resistant population emerges. Whole genome sequencing (WGS) is then employed to identify the genetic basis of resistance. IVIEWGA can inform drug discovery in a number of ways, providing information regarding the molecular targets, resistance mechanisms and resistance potential of compounds in development. However, this approach is not effective for all compounds, specifically those for which resistance cannot be evolved in culture. In terms of drug discovery, these resistance-refractory compounds are highly prized since their low propensity for resistance *in vitro* is considered to foreshadow slow clinical resistance. Understanding the mechanism(s) of action and/or molecular targets of resistance-refractory compounds to facilitate their development is of the highest scientific value. Thus, alternative strategies to support target deconvolution must be found.^9^

Here, we describe comprehensive studies to determine the molecular targets of the resistance-refractory compound MMV022224 (also known as TCMDC-132409). The antimalarial potential of MMV022224 was initially recognized through screening of GlaxoSmithKline’s (GSK) 2 million compound collection against asexual blood stage (ABS) *P. falciparum* . MMV022224 was subsequently designated as a rapid parasite killer and demonstrates activity against multiple stages (blood, liver and sexual) of the parasite lifecycle.^10,11^ Multiple attempts to evolve resistance to MMV022224 using a variety of strategies have proven unsuccessful. Thus, to determine the molecular target(s) of this promising antimalarial and to understand the basis of its low propensity for resistance we have employed two orthogonal chemical proteomics strategies, namely chemical pulldown and thermal proteome profiling (TPP). These orthogonal approaches identify the essential *Plasmodium* kinase, protein kinase 6 (*Pf*PK6),^12^ as a potential target of MMV022224, while also revealing that the compound demonstrates a degree of promiscuity through interaction with additional kinases. The role of poly pharmacology in this compound’s overall potency, stage specificity and low propensity for resistance is discussed. We employed the same methodologies to profile the structurally related azaindole, TCMDC-135051, an established inhibitor of the cyclin-dependent like protein kinase *Pf*CLK3.^13^ Our data confirms that TCMDC-135051 is a relatively selective inhibitor of *Pf*CLK3 thus demonstrating that selective inhibition of specific kinases within the *Plasmodium* kinome is achievable. Collectively, these studies illustrate the utility of chemical proteomics approaches to identify the potential targets of resistance refractory compounds and to reveal the full spectrum of interacting target proteins.

## Results

### MMV022224 Is a Potent Inhibitor of **P. falciparum* In Vitro* with Low Resistance Potential

Profiling of MMV022224 (Figure 1A) confirmed submicromolar potency against ABS and liver stage parasites (EC_50_ values of 390 and 800 nM, respectively).^11^ The ability to rapidly reduce parasite burden is a critical factor for an effective antimalarial since rapid drug action can prevent severe illness and limit the emergence of drug resistance. The speed of action of MMV022224 was assessed using an *in vitro* parasite viability assay.^14^ In this analysis, MMV022224 was designated a fast-acting compound with a comparable profile to the mainstay antimalarial chloroquine.^11^

**Figure 1:**
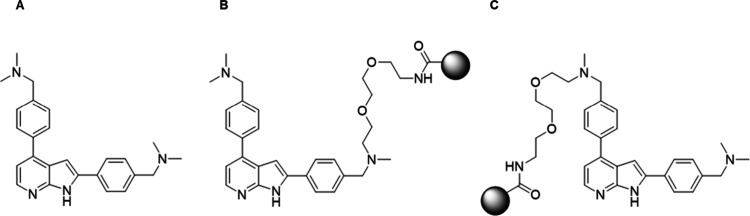
Structure of MMV022224 (A) and associated pulldown probes (B, C).

IVIEWGA is a common and highly effective route toward antimalarial target identification.^8,15^ However, multiple previous attempts to generate MMV022224-resistant cell lines through a variety of strategies failed. We then tried to generate resistant mutant parasites using Dd2Polδ, a mutator Dd2 parasite line with defective proof-reading activity following replacement of D308 and E310 catalytic residues of *P. falciparum* DNA polymerase δ, with alanine. This impaired proof-reading activity endows Dd2Polδ with a 5- to 10-fold higher mutability rate in the presence of a drug compared to Dd2. Parasites were cleared within the first 8–10 days of drug pressure in all three cultures. However, neither single-step continuous pressure at 3× EC_50_ nor ramping selection pressure strategies yielded recrudescent MMV022224-resistant parasites during the 60 days of the study.

To determine if resistance mechanisms to current antimalarial drugs can confer cross-resistance to MMV022224, the compound was screened against the Antimalarial Resistome Barcoding (AReBar) pooled library of *P. falciparum* transgenic cell lines.^16,17^ The AReBar library comprises 53 barcoded parasite lines bearing mutations in established antimalarial drug targets. The library was screened with MMV022224 for 14 days at a concentration equivalent to 3× its EC_50_ value (Figure S1, Table S1). In parallel, selections were carried out with DSM265, known to target *P. falciparum* dihydroorotate dehydrogenase (*Pf*DHODH).^18^ Previous studies have shown that *P. falciparum* expressing the yeast homologue of DHODH (yDHODH) are resistant to inhibitors targeting *Pf*DHODH. As expected, DSM265 selection of the library led to the recrudescence of parasites expressing yDHODH and the PfDHODH-C276Y mutant (Figure S1B). In contrast no viable parasites or enriched barcode profiles were detected following library selection with MMV022224. Collectively, these data suggest that the mechanism(s) of action and mechanism(s) of resistance of MMV022224 are likely distinct from those interrogated within the library (Table S1). It should be noted that compounds with a low resistance potential and acting via potentially unique mechanisms of action are highly prized in antimalarial drug discovery.

During the trophozoite stage, *P. falciparum* parasites extensively degrade host red blood cell hemoglobin, both to generate amino acid precursors required for protein synthesis and to clear intracellular space for parasite growth and development. Haem is produced as a toxic byproduct in this process that must be further metabolized by the parasite to avoid self-toxicity. This is achieved via the haem detoxification pathway located in the acidic digestive vacuole where cytotoxic haem is incorporated into an inert crystalline compound known as hemozoin. Multiple clinically used and experimental antimalarial compounds are known to inhibit hemozoin formation, to such an extent that this can be considered a promiscuous albeit selective mechanism of action within the parasite. To determine if inhibition of hemozoin formation plays any role in the phenotypic activity of MMV022224, we assessed the propensity of this compound to inhibit β-hematin (synthetic hemozoin) formation *in vitro* using a biomimetic cell-free detergent-based assay.^19–21^ While chloroquine (positive control), an established inhibitor of this process, inhibited extracellular β-hematin formation with an IC_50_ value of 24 ± 1 μM, MMV022224 failed to impact β-hematin formation at concentrations up to and including 200 μM (Figure S2). This suggests that inhibition of hemozoin formation does not contribute significantly to the mechanism of action of MMV022224.

### MMV022224 Interacts with Multiple *P. falciparum* Kinases Including *Pf*PK6

Since IVIEWGA failed to provide information regarding either the molecular target(s) or mechanism(s) of action of MMV022224, alternative target identification strategies were pursued. Chemical pulldown is a powerful affinity-based method to identify the protein targets of bioactive compounds.^22,23^ Commonly, derivatives of the compound of interest are immobilized onto a solid support to enrich ligand-binding proteins from cell lysate. Specific and nonspecific binding to resin can be discriminated by carrying out pulldowns in the presence of competition with free compound. Targets specifically interacting with the immobilized derivatives are then identified and quantified by mass spectrometry (MS). In this format (Figure 2A^22^), chemical pulldown represents an unbiased route to identify interacting proteins, with the potential to identify primary targets as well as potential secondary targets.

**Figure 2:**
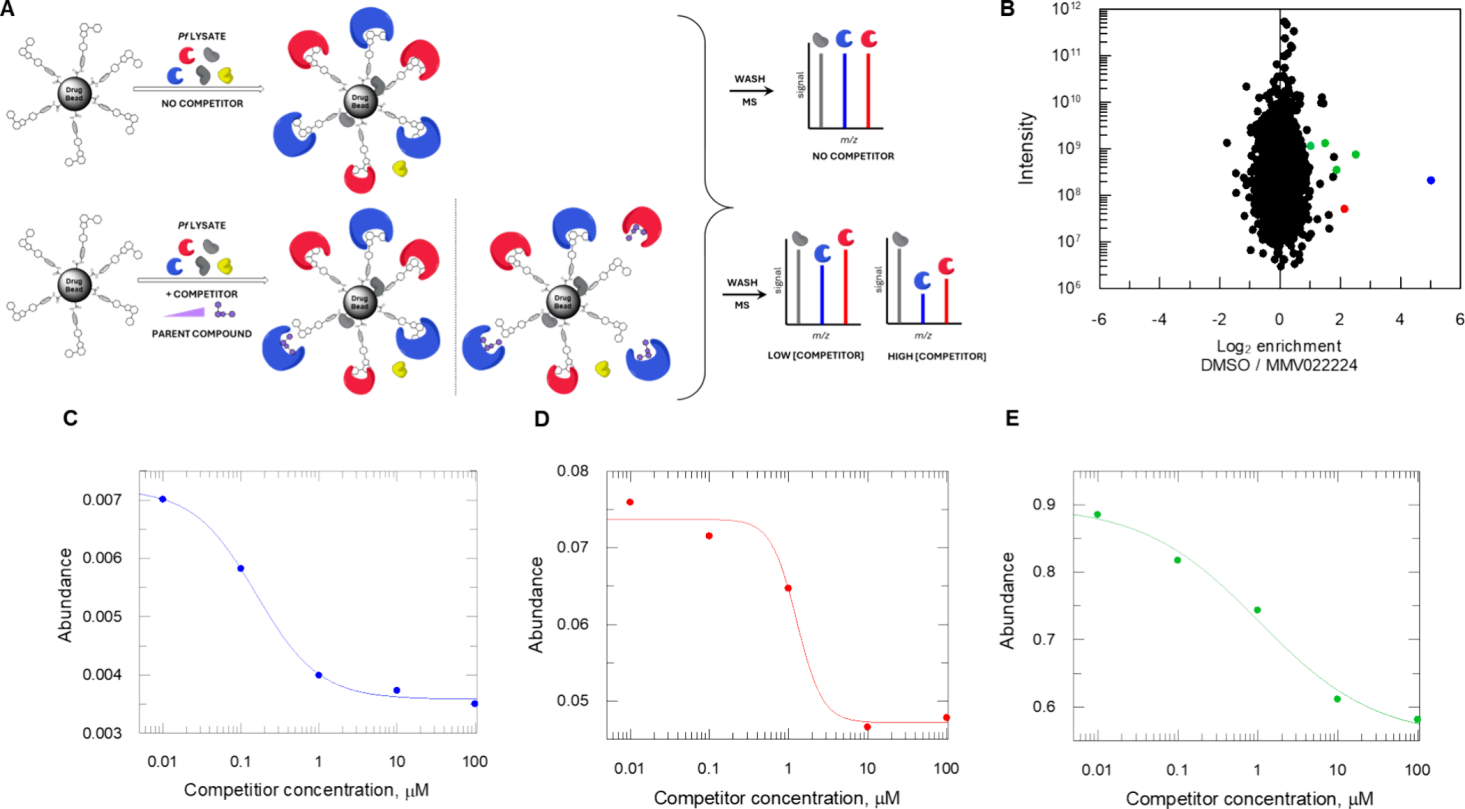
Chemical pulldown with MMV022224 probe 1. (A) Schematic representation of chemical pulldown strategy. (B) Differential binding of *P. falciparum* lysate-derived proteins to “low load” resin-bound probe 1 in the presence of free MMV022224 (100 μM) or DMSO. PK6 highlighted in blue, CLK3 highlighted in red and enriched proteins bearing kinase domains highlighted in green. Relative abundance of PK6 (C), CLK3 (D), and PI3/4K (E) in the presence of dose dependent competition with MMV022224. IC_50_ values of 0.16 ± 0.03, 1.3 ± 1.3, and 1.0 ± 0.8 μM were established for PK6, CLK3 and PI3/4K, respectively.

Attachment of derivatives to resin is achieved through addition of a linker moiety, usually a polyethylene glycol (PEG) chain terminating in a protected amine, to the bioactive compound. The position of linker attachment can be guided by knowledge of the molecule’s structure–activity-relationship (SAR). When this information is not available, two or more distinct, chemically tractable vectors are selected, linker analogues prepared and then profiled to assess the impact of the modifications on potency. In the absence of supporting SAR information, Boc-protected versions of two linker analogues (MMV022224 probes 1 and 2 shown in Figure 1B,C, respectively) were profiled, alongside the parent compound, against ABS parasites. As expected, attachment of the PEG linker marginally decreased the potency of probe 1 relative to the original parent compound (EC_50_ values of 241 ± 13 and 217 ± 11 nM for probe 1 and MMV022224, respectively) (Table 1). However, attachment of the PEG linker at the 4-position of the azaindole surprisingly enhanced potency ∼1.6-fold (EC_50_ value: 138 ± 5 nM). Since the potency of linker 1 tracked more closely with MMV022224 and bearing in mind that increased potency may indicate that probe 2 is interacting with different or additional molecular targets, probe 1 was progressed to pulldown studies.

**Table 1:** Collated EC_50_ Values against *P. falciparum* (3D7) Asexual Blood Stage

compound	EC_50_ values^a^, nM	fold shift (relative to parent)	biological replicates
MMV022224	217 ± 11		5
MMV022224 – probe 1	241 ± 13	1.1	4
MMV022224 – probe 2	138 ± 5	0.6	4
TCMDC-135051	197 ± 3		6
TCMDC-135051 – probe 1	352 ± 14	1.8	6
TCMDC-135051 – probe 2	275 ± 17	1.4	6

a EC_50_ values represent the weighted mean ± SD.

The masked amine on probe 1 was deprotected and immobilized onto NHS (*N*-hydroxysuccinimide)-activated magnetic resin. Pulldowns were carried out with this resin in the presence and absence of competition from MMV022224 (100 μM), leading to selective enrichment of several proteins (Figure 2B, Table S2). Notably, three proteins were considered significantly enriched (>2 log_2_ fold change in the presence of competition). All three were identified as kinases and classified as indispensable/essential in *P. falciparum* ABS parasites via the PiggyBac transposon mutagenesis screen.^12^ The most highly enriched protein was *P. falciparum* protein kinase 6 (*Pf*PK6; PF3D7_1337100), recently identified as a potential antimalarial drug target.^24^ The second target candidate identified is annotated as a phosphatidylinositol 3′, 4′-kinase (*Pf*PI­(3,4)­K, PF3D7_0311300.1), involved in lipid metabolism. Based on in depth sequence analysis, this little studied kinase most likely functions a class II PI4K, phosphorylating the phosphatidylinositol ring at the 4′-position to produce phosphatidylinositol 4′ phosphate.^25^ The remaining kinase significantly enriched by probe 1 drug beads was the promising antimalarial drug target cyclin-dependent like protein kinase 3 (*Pf*CLK3, PF3D7_1114700.1), reported to play a role in the phosphorylation and assembly of components of the parasite RNA spliceosome.^26^ *Pf*CLK3 was first identified as a viable drug target in malaria following comprehensive drug target deconvolution studies with TCMDC-135051 (Figure 4A), a potent antimalarial demonstrating activity against blood-, liver- and transmissible sexual stages of the parasite.^26^ TCMDC-135051 has since entered a drug development program aimed at generating a clinical candidate with multistage potential.^27^

It should be noted that probe 1 drug beads enrich several additional *P. falciparum* kinases, albeit at lower levels of enrichment (log_2_ between <2 and >0.5) than the primary hits (Figure 2B, Table S2). These data suggest that while MMV022224 selectively interacts with a small cohort of genetically essential *Plasmodium* kinases, this 7-azaindole may also demonstrate a degree of promiscuity, binding with lower affinity to multiple additional parasite kinases.

### Prioritizing MMV022224 Targets Based on Affinity

To generate more nuanced information from our pulldowns with probe 1, we repeated the pulldowns in the presence of dose-dependent competition with the parent at concentrations ranging from 0.01–100 μM. This variant of chemical pulldown can provide invaluable information regarding the relative binding affinities of individual targets and help prioritize validation studies. Pulldowns in competition with varying concentrations of MMV022224 identified the same three targets as in our initial experiment. Once again *Pf*PK6 was identified as the top hit in this pulldown demonstrating an IC_50_ value of 161 ± 30 nM in competition with MMV022224 (Figure 2C, Table S2), which aligns with the *in vitro* potency of the compound against ABS parasites. *Pf*PI­(3,4)K and *Pf*CLK3 also responded in a dose dependent manner to competition from the parent compound with IC_50_ values of 1.3 and 1.0 μM, respectively (Figure 2D,E,Table S2). Collectively, these data confirm *Pf*PK6 binds to MMV022224 with the highest affinity, while also identifying *Pf*PI­(3,4)K and *Pf*CLK3 as high affinity binders.

### Further Evidence of MMV022224 Target Engagement

To provide further confirmation of the binding targets of MMV022224, we employed isothermal proteome profiling (iTPP),^28^ a rationalized version of standard thermal proteome profiling (TPP).^29^ Both iterations of TPP are based on the principle that binding of a drug to its protein target can alter the thermal stability of that target. In the iTPP assay, the relative abundance of proteins in cell lysates is monitored at a single temperature in the presence and absence of test compounds. Increased abundance of a specific protein in the presence of a compound is indicative of thermo-stabilization of the target due to a direct interaction with the ligand.

In the current study, whole cell lysates of *P. falciparum* 3D7 ABS parasites were prepared and exposed to MMV022224 at 10× their respective EC_50_ values or DMSO as a vehicle control for 30 min. Our previous studies illustrated that the *P. falciparum* proteome has a *T*_m_ (temperature at which 50% of all proteins are denatured) of ∼45 °C. To maximize the chances of identifying proteins stabilized in the presence of compound, treated and control lysates were incubated at 48 °C. Following incubation, insoluble (denatured) proteins were removed, and the abundance of remaining soluble proteins in each treated and control sample was determined by quantitative mass spectrometry. Proteins demonstrating a > 1.5-fold shift in abundance (increase or decrease) in the presence of drug were considered as “hits” and putative targets of the compound. In keeping with our pulldown data, *Pf*PK6 was identified as the top hit and most compelling candidate target of MMV022224, with the relative abundance of this kinase increasing considerably in the presence of compound across two biological replicates (Figure 3A–C, Table S2). These data provided further evidence to support *Pf*PK6 as a potential target of this 7-azaindole. Across both iTPP biological replicates (Figure 3C), *Pf*CLK3 was consistently identified as the second most stabilized protein in the presence of MMV022224, however, in contrast to our pulldown studies, *Pf*PI­(3,4)K was not stabilized despite being successfully detected in the lysate and exhibiting a *T*_m_ within the scrutinized temperature window (∼43 °C). However, it should be noted that an extensive search of the literature failed to identify a single instance where direct physical binding of *Pf*PI­(3,4)­K inhibitors to their intracellular target had been demonstrated either by CETSA or TPP. This may suggest that *Pf*PI­(3,4)­K is not compatible with these biophysical techniques and failure to identify this lipid kinase in our data set does not exclude it as a potential target of MMV022224.

**Figure 3:**
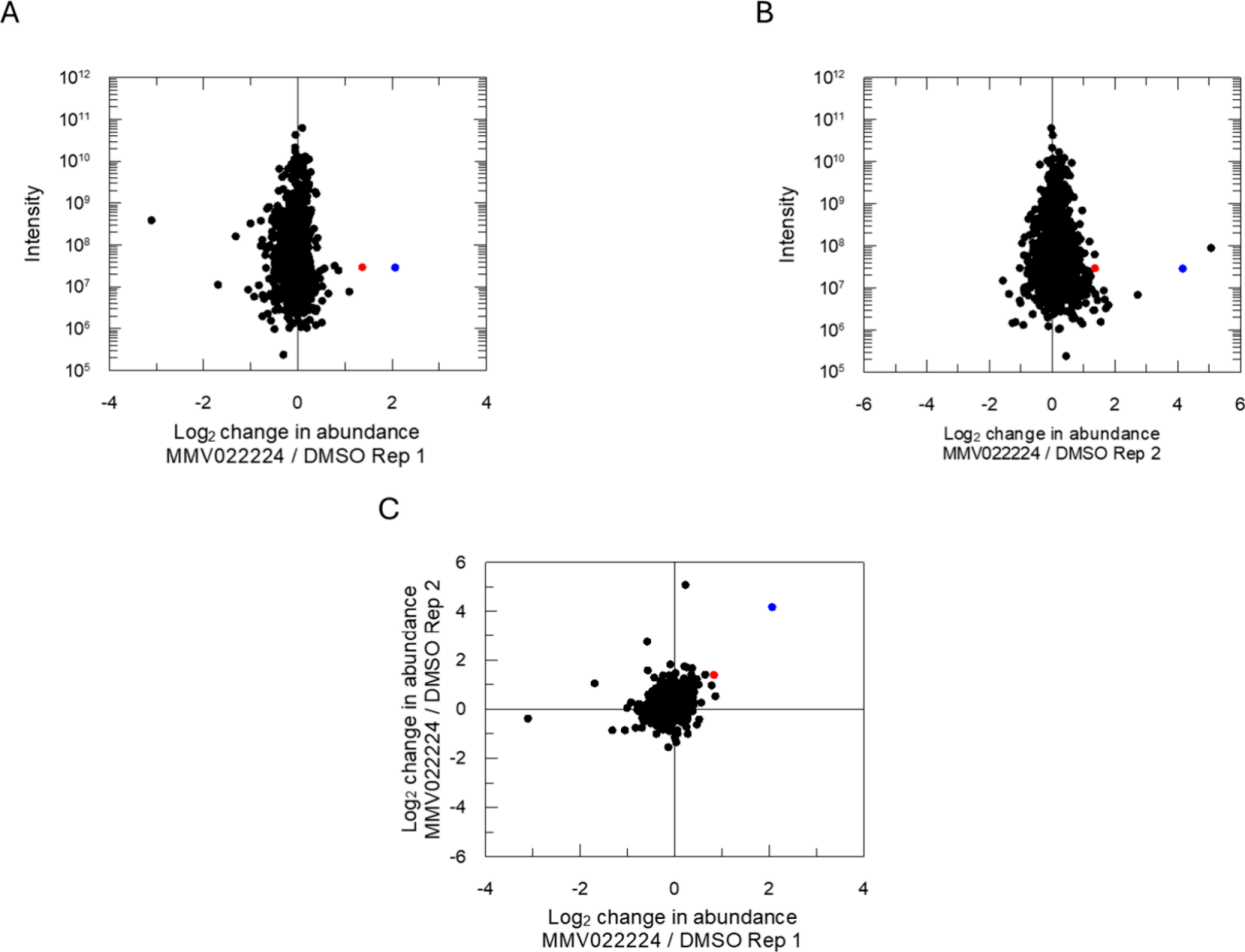
Isothermal TPP analysis of the *P. falciparum* proteome ± MMV022224 treatment. Plots show protein abundance log_2_ fold change between compound-treated and untreated lysates subjected to thermal shock at 48 °C. Lysates exposed to MMV022224 (10 μM). Data are sorted by protein total intensity on the *y*-axis. Only proteins identified with >2 unique peptides shown. *Pf*PK6 is indicated in blue and *Pf*CLK3 indicated in red. Data from biological replicates 1 and 2 shown in panels A and B respectively, merged data shown in panel C.

### Inhibition of *Pf*PK6 Enzyme Activity

As the top hit in both our iTPP and chemical pulldown studies, we next assessed if MMV022224 binding to *Pf*PK6 translated to inhibition of enzymatic activity. *Pf*PK6 was recombinantly expressed, purified and assayed via a previously established ADP-glo assay.^24^ In this assay, the IC_50_ value of MMV022224 was determined as 800 ± 100 nM compared to 500 ± 200 nM for the positive control Staurosporine (Table 2). Further, dose-dependent inhibition of *Pf*PK6 phosphorylation of a generic substrate (histone H1) was observed in a radiometric kinase assay using γ^32^P-ATP (Figure S3). We also tested MMV022224 probe 1, which exhibited a 1.75-fold reduced potency against *Pf*PK6 compared to the parent compound (IC_50_: 1400 ± 600 nM). While these data confirm MMV022224 is an inhibitor of *Pf*PK6, this level of enzymatic inhibition is not entirely consistent with the phenotypic potency of this compound and may suggest that *Pf*PK6 is not the sole target driving antimalarial activity. Indeed, a scenario where MMV022224’s antimalarial activity was achieved via inhibition of more than a single molecular target would also be consistent with the failure to generate MMV022224 resistance through IVIEWGA.

**Table 2:** Potency of Test Compounds in *PfPK6* Enzymatic Assays

compound	*Pf*PK6 inhibition (IC_50_, μM)^a^
MMV022224	0.8 ± 0.1
TCMDC – 135051	2.7 ± 0.6
MMV022224– probe 1	1.4 ± 0.6
TCMDC-135051– probe 1	3 ± 0.9
staurosporine	0.5 ± 0.2

a IC_50_ values represent the mean ± SD of three independent experiments.

### Impact of *Pf*PK6 Knockdown on Viability and Potency

We next assessed the impact of *Pf*PK6 conditional knockdown on parasite viability and MMV022224 susceptibility. Conditional knockdown of *Pf*PK6 was achieved using the previously described *Pf*DOZI-TetR system.^30,31^ This system enables translational regulation by incorporating tetracycline repressor (TetR)-binding aptamer sequences upstream or downstream of the *Pf*PK6 open reading frame. *Pf*DOZI-TetR binds to the aptamers, resulting in modest *Pf*PK6 translational repression in parasites bearing aptamers in the 3′ position (Figure S4A) and stronger repression in parasites bearing aptamers in the 5′ and 3′ position (Figure S4B). The addition of anhydrotetracycline (aTc) relieves repression by preventing *Pf*DOZI-TetR from binding to the aptamers. After growth in the absence of aTc for 72 h and under both repression systems (3′ only and 5′ plus 3′), levels of *Pf*PK6 fell below limits of detection via Western blotting. While more modest repression of *Pf*PK6 expression had little impact on parasite growth (Figure S4C,D), strong repression led to a severe growth defect indicating that this kinase is essential for survival of ABS parasites (Figure S4E,F).

ABS parasites subjected to *Pf*PK6 knockdown under modest and stringent conditions were assessed for their susceptibility to MMV022224 (Figure S4G,H). Under modest knockdown conditions, we did not see the expected increased susceptibility to MMV022224, that would be indicate a standard target-compound relationship. While under stringent knockdown conditions, parasites became 1.3-fold less susceptible to MMV022224. Neither of these observations are consistent with our initial hypothesis that *Pf*PK6 is the primary target of MMV022224 but do not rule out *Pf*PK6 as one of a group of targets driving the phenotypic activity of this azaindole. It should also be noted based on this data we cannot exclude the possibility that *Pf*PK6 plays no substantive role in the mode of action of MMV022224, despite the evident high affinity target-compound binding interaction.

### Exploring the Broader Target Landscape of TCMDC-135051 via Chemical Proteomics

TCMDC-135051, an azaindole demonstrating potent antimalarial activity across a range of parasite stages, was also identified as a potent antimalarial through screening of GSK’s 2 million compound collection.^26,32^ This compound, known to target *Pf*CLK3,^26^ shares striking structural similarity with MMV022224 (Figure 4A). Based on this structural similarity and the confirmed interaction between MMV022224 and the acknowledged target of TCMDC-135051, *Pf*CLK3, we decided to profile the broader target landscape of TCMDC-135051 through chemical proteomics strategies.

**Figure 4:**
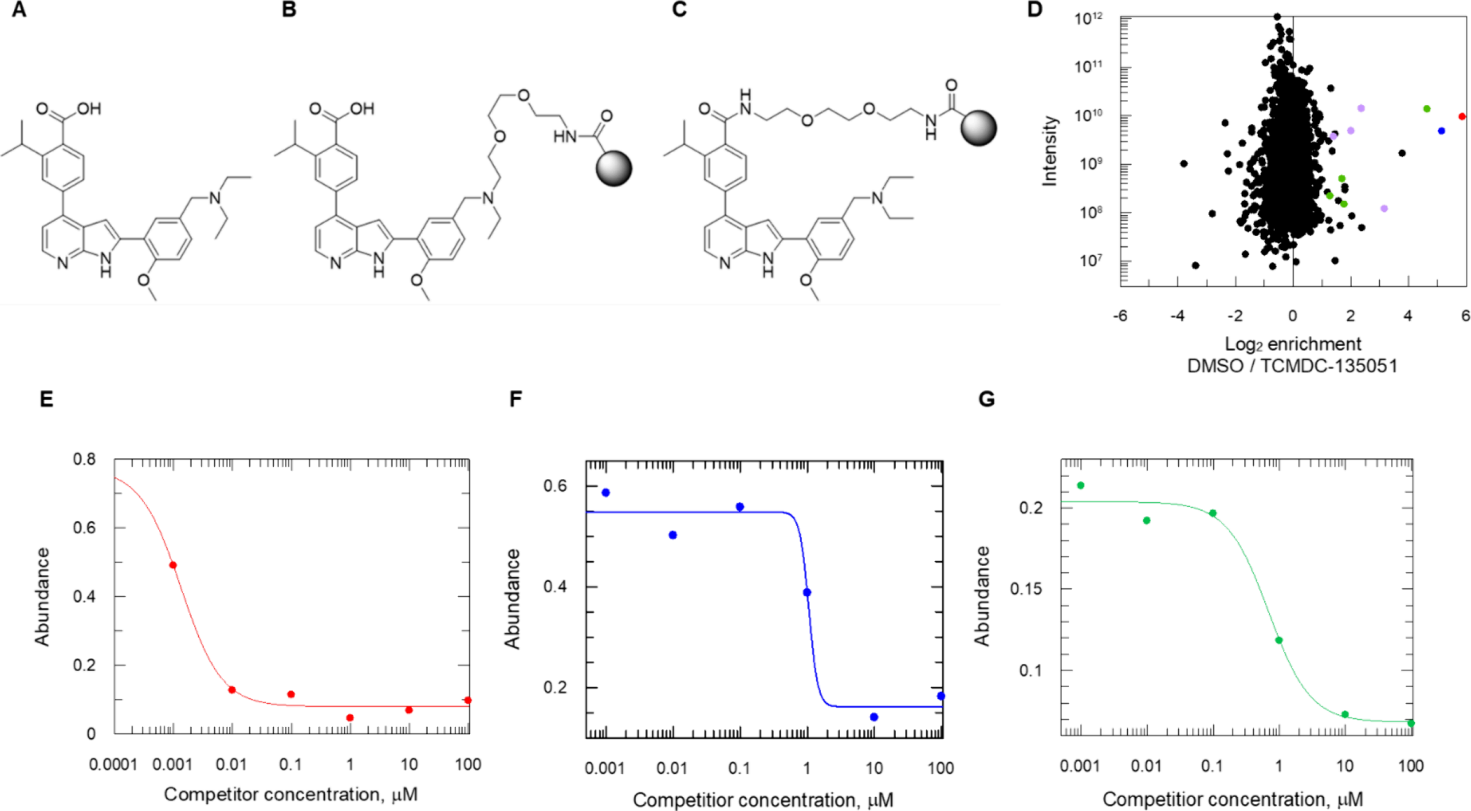
Chemical pulldown with TCMDC-135051 probe 2. (A) Structure of TCMDC-135051 and associated pulldown probes 1 (B) and 2 (C). (D) Differential binding of *P. falciparum* lysate-derived proteins to “low load” resin-bound probe 1 in the presence of free TCMDC-135051 (100 μM) or DMSO. *Pf*PK6 highlighted in blue, *Pf*CLK3 highlighted in red, enriched proteins bearing kinase domains highlighted in green and casein kinases highlighted in lilac. Relative abundance of *Pf*CLK3 (E), *Pf*PK6 (F), and nucleoside diphosphate kinase (G) in the presence of dose dependent competition with TCMDC-135051. IC_50_ values of 0.0013 ± 0.0017, 1.08 ± 0.6, and 1.2 ± 1.5 μM were established for *Pf*CLK3, *Pf*PK6, and nucleoside diphosphate kinase, respectively.

To explore two distinct vectors for linker attachment, linker analogues of TCMDC-135051 were synthesized with PEG linkers attached on each of the two benzene moieties (Figure 4B,C). Profiling of both linker analogues against ABS parasites confirmed that they retained activity (Table 1), however, linker 1 was selected for pulldown studies since it retained the carboxylic acid group previously deemed as important for *Pf*CLK3 binding.^27^ The masked amine of TCMDC-135051 probe 1 was deprotected and the probe was immobilized on NHS-activated Sepharose resin. As described for MMV022224, pulldowns with TCMDC-135051 probe 1 were carried out in competition with a single concentration (100 μM, Figure 4D, Table S3) and dose-dependent competition with the parent inhibitor (Figure 4E–G and Table S3 and Figure S4). As expected, in both pulldown strategies the established molecular target *Pf*CLK3 was identified as the top hit, with an IC_50_ value of 0.001 ± 0.001 μM in our dose-dependent competition study (Table S3, Figure 4E). *Pf*PK6 was identified as the next highest affinity binder of TCMDC-135051 with an IC_50_ value of 1.08 ± 0.6 μM, with another genetically essential kinase, nucleoside diphosphate kinase, in third (IC_50_ value: 1.2 ± 1.5 μM) (Figure 4F,G). Notably, of the 17 remaining proteins significantly enriched by TCMDC-135051 probe 1, 5 were identified as class II casein kinases (both human and *Plasmodium*)^33,34^ (Table S3 and Figure S5). While interaction with casein kinases is unlikely to play any significant role in driving the antimalarial activity of TCMDC-135051, this association may be worth monitoring as a potential toxic liability of analogues emerging from TCMDC-135051-related drug discovery programs.^27,35^

iTPP was again used as a secondary, unbiased approach to identify the molecular target(s) of TCMDC-135051. Across two biological replicates *Pf*CLK3 was by far the most thermally stabilized protein in the presence of the parent compound (Figure 5 and Table S4). *Pf*PK6 was identified as the second most stabilized target albeit significantly less stabilized than *Pf*CLK3.

**Figure 5:**
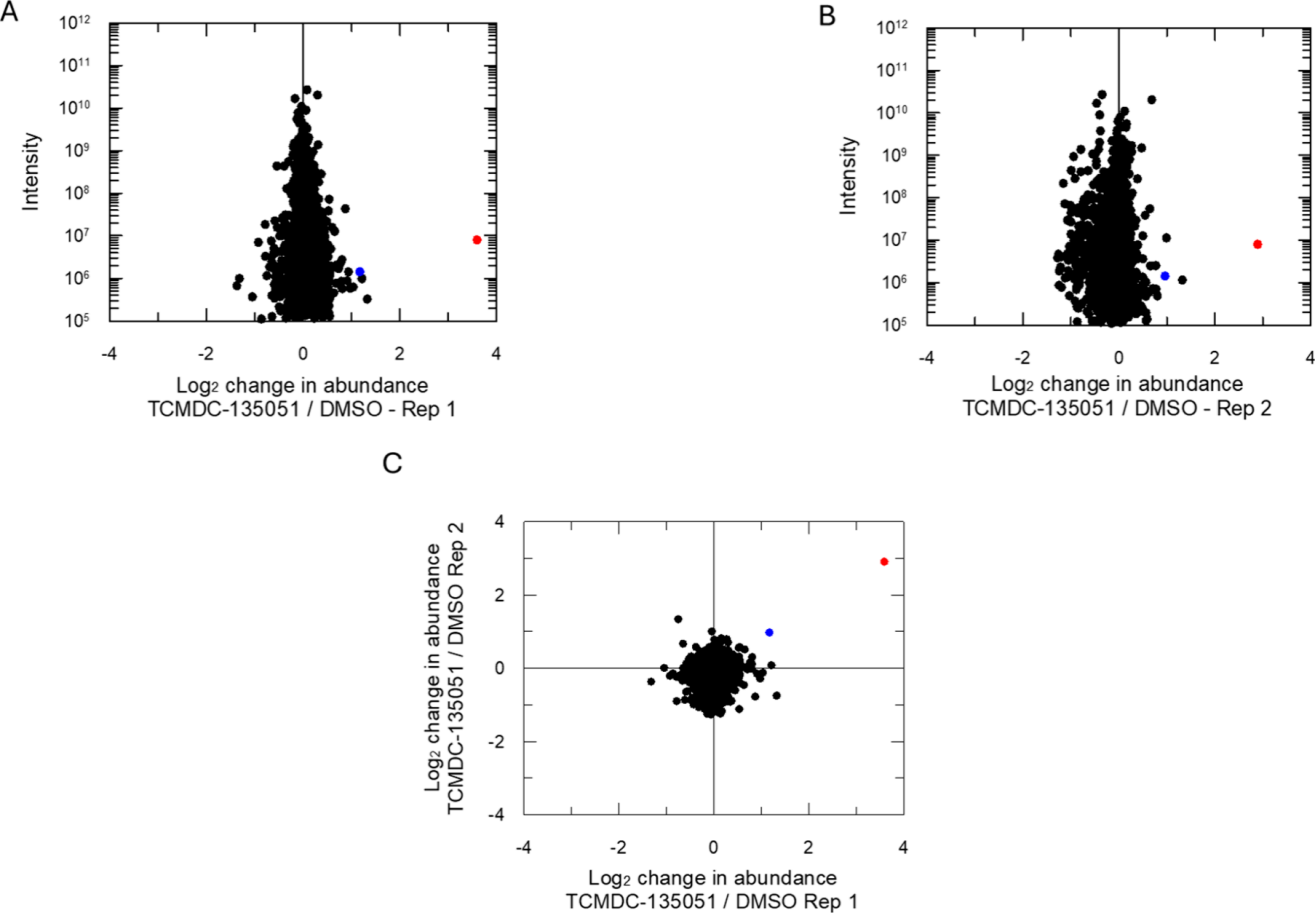
Isothermal TPP analysis of the *P. falciparum* proteome ± TCMDC-135051 treatment. Plots show protein abundance log_2_ fold change between compound-treated and untreated lysates subjected to thermal shock at 48 °C. Biological replicates 1, 2, and combined hits shown in panels (A), (B), and (C), respectively. Lysates exposed to TCMDC-135051 (10 μM). Data are sorted by protein total intensity on the *y*-axis. Only proteins identified with >2 unique peptides shown. *Pf*PK6 is indicated in blue and CLK3 indicated in red.

Screening of TCMDC-135051 against the previously described AReBar library further confirmed *Pf*CLK3 as its predominant molecular target (Figure S1B and Table S1). Selection of the library with this 7-azaindole (3× EC_50_) led to outgrowth of parasites bearing the resistance-conferring H259P mutation in *Pf*CLK3.^26^ Indeed, standard growth inhibition assays with the individual *Pf*CLK3^H259P^ cell line confirmed that it was 6-fold less susceptible to TCMDC-135051 than the parental Dd2 line (Table S7). In contrast, this transgenic cell line remained just as susceptible to MMV022224 as Dd2. These data confirm TCMDC-135051 as a potent and highly selective inhibitor of *Pf*CLK3, and while this compound selectively binds to *Pf*PK6, there is no direct evidence to support this interaction playing a significant role in its phenotypic activity.

## Discussion

In our current study, unbiased approaches were employed to explore the potential target environment of the promising antiplasmodial MMV022224. Understanding the mechanism of action of this 7-azaindole was prioritized since it had a low propensity for resistance *in vitro*, was designated a rapid parasite killer and appeared to act via a potentially unique mechanism, all properties that are highly prized in antimalarial drug discovery. A combination of chemical pulldown and isothermal proteome profiling revealed that MMV022224 interacts with a range of *Plasmodium* kinases but binds to *Pf*PK6 with highest affinity. Subsequent enzymatic and molecular studies confirmed that while this compound inhibits *Pf*PK6, its phenotypic activity and apparent high barrier to resistance *in vitro* may be due to interaction and inhibition of multiple kinases. Employing the same approaches, we also profiled the structurally related azaindole TCMDC-135051. In contrast to MMV022224, TCMDC-135051 proved to be a potent and relatively selective inhibitor of *Pf*CLK3, while *Pf*PK6 was a consistently enriched potential additional target. In keeping with these observations, a recently reported virtual screen of the Tres Cantos Antimalarial Set^14^ against the *Pf*PK6 predicted MMV022224, but not TCMDC-135051, to be a *Pf*PK6 ligand.^36^

The jury is still out on whether *Pf*PK6 is a viable or exploitable antimalarial drug target. We contend that in the absence of demonstrably selective inhibitors, *Pf*PK6 remains to be robustly chemically validated. Undoubtedly, a number of extremely potent *Pf*PK6 inhibitors have been identified through enzymatic^24^ and target binding displacement *in vitro* assays.^37^ Specifically, a series of type II kinase inhibitors demonstrating single digit nM potency against *Pf*PK6 in the KinaseSeeker split luciferase assay have recently been reported.^38^ However, not all of these inhibitors were active against ABS *P. falciparum* and there was relatively little correlation between enzymatic versus antiparasitic activity. The authors acknowledge that the best performing inhibitors from this cohort are likely to exert their antiplasmodial effects via polypharmacology. Similarly, a 2-aminobenzimidazole inhibitor of *Pf*PK6 with potent *in vitro* and *in vivo* antimalarial activity was reported.^24^ In depth mode of action studies revealed that this compound also targets hemozoin formation. Indeed, *Pf*PK6 was one of the most frequently inhibited kinases from a 5 kinase enzyme panel screened against the Tres Cantos Antimalarial Set (TCAMS) library, perhaps suggesting that polypharmacology may be inexorably linked to this target.^39^ Our conditional knockdown studies indicate that parasite viability is only impacted upon *Pf*PK6 knockdown under the most stringent conditions. These are entirely consistent with reports that ABS parasites remain viable with less than 5% of wild type *Pf*PK6 levels.^24^ Thus, it remains to be seen if a small, drug-like inhibitor selectively and solely targeting *Pf*PK6 can achieve the level of inhibition required to drive antimalarial activity. Beyond drug discovery, potent and selective inhibitors of *Pf*PK6 would be invaluable tools to better define the physiological function of this kinase.

As discussed, our studies indicate a close association between *Pf*CLK3 and *Pf*PK6 and suggest that relatively minor changes in inhibitor design can switch affinity and/or binding preference between these two kinase targets. This raises the possibility of designing inhibitors that specifically target both kinases. It would be interesting to see if such a therapeutic could combine the impressive properties and potencies demonstrated by compounds that target *Pf*CLK3 ^26,27,35^ with a reduced propensity for resistance and lower efficacious dose required. Design of dual- or multitarget kinase drugs is certainly not a new concept and strategies to leverage the acknowledged cross-reactivities of small molecule kinase inhibitors in this way are well understood.^40^ Indeed, the discovery that the human mTOR inhibitor sapanisertib targets *Plasmodium* phosphatidylinositol 4-kinase type III β (PI4Kβ) and cyclic guanosine monophosphate (cGMP)-dependent protein kinase (PKG),^41^ led to suggestions that PI4Kβ-PKG dual targeting antimalarials may be feasible. In this instance, the possibility of inhibiting *Pf*PI4Kβ, a target associated with rapid parasite clearance in the clinic and *Pf*PKG shown to induce slow parasite death when inhibited *in vitro* was deemed as highly advantageous. While *Pf*CLK3 inhibition mediates rapid killing of asexual liver- and blood-stage *P. falciparum* and blocks gametocyte development,^13^ further studies will be required to determine the precise parasite death kinetics and stage specificity associated with selective inhibition of *Pf*PK6. This assessment could be achieved using the “bump and hole” approach pioneered by Shokat and colleagues.^42^ Here, bulky kinase gate-keeper residues are replaced with smaller amino acids, leaving the mutated kinase uniquely sensitive to bumped kinase inhibitors. Selective inhibition of *Pf*PK6 in this way, combined with administration of a selective *Pf*CLK3, could be an effective way to explore the impact of dual inhibition of these two genetically essential kinases.

Key physicochemical properties of MMV022224 were measured to assess its suitability as a starting point for an antimalarial drug discovery program (Table S8). Although the optimal property profile will depend upon the intended route of administration (oral vs long-acting injectable), efforts to improve selectivity and metabolic stability should be prioritized if this compound were to enter such a program. However, we acknowledge that developing an inhibitor whose mechanism of action may be driven by polypharmacology may be extremely challenging.

Emerging drug resistance is a major threat to malaria control efforts around the world and developmental compounds with a reduced propensity for resistance or, as in the case of MMV022224, no apparent route to resistance *in vitro*, are prioritized. The ultimate goal is to identify, then develop drugs against an immutable drug target. Whether such a target exists in *Plasmodium spp*. remains to be seen. Target deconvolution is crucial to this endeavor; however, resistance refractory compounds are often the most challenging to study. Since IVIEWGA is ineffective in these cases, alternative strategies are required. Our current studies demonstrate the value of approaches such as competitive chemical pulldown and thermal proteome profiling to facilitate antimalarial drug target deconvolution, particularly for resistance-refractory compounds. Indeed, such chemical proteomics strategies can rightly be considered the gold standard in drug target identification since they can reveal the full repertoire of a drug’s targets, often in a single experiment, as well as providing an indication of individual target binding affinity. These powerful, proteome-wide technologies should be considered an important and valuable part of the antimalarial drug target deconvolution toolkit.

## Methods

### Ethics Statement

At Dundee, parasites were cultured in fresh human erythrocytes obtained with ethical approval from anonymous healthy donors, with informed written consent as part of the recruitment process, from the Scottish National Blood Transfusion Service (SNBTS). The use of erythrocytes was approved by the University of Dundee Schools of Medicine and Life Sciences Research Ethics Committee (Approval reference: 21/39). At Columbia, deanonymized, pooled blood was purchased from the New York Blood Centre, under a protocol approved by the Columbia University Institutional Reviewed Board that deemed this as not human subjects’ research.

### *P. falciparum* Cell Culture

ABS *Plasmodium falciparum* (3D7) were cultured as previously described.^44^ Briefly, parasites were grown at 3% hematocrit and maintained between 0.5 and 5% parasitaemia in erythrocytes (Scottish National Blood Transfusion Service) supplemented with complete malaria media (CMM; RPMI 1640 media supplemented with 25 mM HEPES, 2 mM glutamine, 5 g/L Albumax II, 12 mM sodium bicarbonate, 11 mM glucose, 0.2 mM hypoxanthine, 20 mg/L gentamicin, pH 7.3). Cultures were cultivated at 37 °C in a humidified atmosphere of 1% O_2_, 3% CO_2_ in a balance of N_2_. Media was exchanged every 24–48 h and fresh erythrocytes added every 48–72 h. When required, parasites were synchronized with two rounds of D-sorbitol treatment, as previously described.^45,46^

### Chemical Synthesis

Details of synthesis of chemical probes and parent compounds used in this study are provided in the Supporting Information.

### *P. falciparum* Drug Sensitivity Assays

A SYBRGreen based assay was used to determine the potency of test compounds and chemical probes used in this study.^46^ Serial dilutions of drug (2-fold) in CMM were set up in 96-well tissue culture plates. Parasites and erythrocytes were added to each well to a final hematocrit of 2.5% and 0.3% parasitaemia in a total volume of 100 μL/well. Mefloquine, 10 μM served as a 100% inhibition control. Plates were incubated for 72 h. Subsequently, SYBRGreen I reagent (3×, Thermo Fisher) in lysis buffer (20 mM Tris-HCl, 5 mM EDTA, 0.16% (w/v) saponin, 1.6% (v/v) Triton X-100, pH 7.9) was added to each well (50 μL) and incubated in the dark at room temperature for a further 3–4 h. Fluorescence (excitation 485 nm, emission 528 nm) was then measured using a Tecan Infinite Pro 200 microplate reader. Dose response curves and concentrations of compound required to inhibit parasite growth by 50% (EC_50_) were calculated using a two-parameter equation in Grafit version 7.0 (Erithacus Software) shown below:

$$y=\frac{100}{1+{\left(\frac{\left[I\right]}{EC50}\right)}^{m}}$$

[*I*] represents the inhibitor concentration, and m is the slope factor. Experiments were performed in, at least, three independent biological replicates, and the data are presented as the weighted mean ± standard deviation.

### Antimalarial Resistome Barcoding Assay (AReBar)

AReBar compound profiling for cross-resistance was performed essentially as previously described.^16,17^ In brief, a panel of 53 barcoded lines representing common targets or modes of resistance (Table S1) was exposed for 14 days to 3 × EC_50_ of MMV0222244, TCMDC-135051, the dihydroorotate dehydrogenase inhibitor DSM265,^18^ or an untreated control. Growth of cultures was monitored by flow cytometry (stained with 1× SYBR Green and 200 nM Mitotracker Deep Red), and parasitaemia adjusted to maintain cultures below 5%. Samples were collected at day 0 and 14, saponin-lysed (0.05% (w/v) saponin), and the barcode amplified. Amplicons were prepared for sequencing using the Oxford Nanopore Technologies (ONT) native barcoding kit 96 (SQK-NBD114.96) and sequenced on an ONT minION Mk1c to measure the relative proportion of each parasite line.

### β-Hematin Assay

A previously described *in vitro*, cell-free, detergent-mediated Nonidet P-40 (NP40) assay was used to identify potential inhibition of β-hematin formation.^19–21^

### Chemical Pulldown: *P. falciparum* Lysate Preparation

Synthesis of all chemical probes and parent compounds used in this study are provided in the Supporting Information. Our detailed protocol for chemical pulldown studies in *P. falciparum* can be found in Smith et al.^22^ Briefly, 2–4 days prior to lysate preparation, unsynchronised cultures were transferred into CellBIND surface hyperflasks (Corning) and the hematocrit reduced to 2%. Media was refreshed once or twice daily until parasitaemia reached between 8–15%. Cells were pelleted by centrifugation (1800*g*, 15 min, RT, brake 2) and erythrocytes lysed by treatment with 0.1% saponin (w/v) in wash buffer (100 mM potassium acetate, 2.5 mM magnesium acetate, 45 mM pH 7.4 HEPES, 250 mM sucrose, 2 mM dithiothreitol [DTT], 15 μM leupeptin). Following 10 min incubation on ice with regular agitation, lysed cells were centrifuged (2800*g*, 8 min, brake 5, 4 °C). Resulting pellets were washed 3 times in wash buffer until the supernatant became clear. Parasites were resuspended in one volume of lysis buffer (wash buffer supplemented with Roche cOmplete EDTA-free protease inhibitor cocktail [1 tablet/20 mL] and 0.5% (v/v) NP40) and lysed by nitrogen cavitation (1500 psi, 60 min, on ice) in a cavitation unit (Parr). Lysate was then centrifuged (20,000*g*, 15 min, 4 °C), the resulting supernatant transferred to a fresh tube and its protein concentration determined by Bradford assay before immediately continuing with the pulldown assay and adjusted to a final concentration of 2 mg protein/mL with lysis buffer.

### Resin Preparation for Chemical Pulldown Studies

MMV022224 and TCMDC-135051 probes (Figure 1 and 4B) were immobilized onto NHS-derived magnetic (Pierce) or NHS-activated Sepharose 4 Fast Flow (Cytivia) resin using previously described methods (magnetic,^47^ Sepharose^22^). Loading levels for each Sepharose drug bead set were determined as previously described^22^ and reported in Results.

### Chemical Pulldown

Lysates were preincubated with 2 mg blank resin capped with ethanolamine (prewashed 2× in dH_2_O followed by 2× in lysis buffer) with rotation for 30 min at 4 °C. The beads were pelleted by centrifugation (10,000*g*, 30 s), the supernatant divided in two and incubated either with 1% DMSO or test compound in 1% DMSO with rotation at 4 °C for 30 min. For single concentration competition pulldowns, lysates were preincubated with 100 μM of the relevant parent compound. For dose–response competition pulldowns, aliquots of lysate were incubated with concentrations of the relevant parent compound ranging from 100 μM to 10 nM (10-fold dilutions). Following incubation, drug-beads (2 mg prewashed beads per sample) were added to treated lysates and rotated at 4 °C for 60 min. Beads were then washed 3× in wash buffer (50 mM Tris-HCl, pH 8.0, 5 mM EDTA, 1 mg/mL BSA, 0.5% [v/v] NP40) then 2× with Tris-buffered saline before being resuspended in 25 μL NuPage loading buffer (25% NuPage LDS buffer, 50 mM DTT in dH2O) and heated to 95 °C for 10 min. Samples were then subjected to electrophoresis on a NuPAGE 4–12% Bis-Tris gel. Once lysate proteins had progressed 1.5 cm into the gel, electrophoresis was stopped and the gel stained with Coomassie quick reagent (NeoBiotech). Proteins were then excised from each lane.

### Chemical Pulldown – Sample Processing, Fractionation, Protein Identification, and Quantitation

All aspects of sample processing, TMT labeling, fractionation by HPLC and LC–MS/MS, and protein identification and quantitation were described previously.^28^ In this instance, samples were separated into 10 fractions. Proteins were identified by searching the MS and MS/MS data for the peptides from the *P. falciparum* strain 3D7 (Plasmo DB version 45, plasmodb.org) and Human proteomes (Uniprot: Accession 9606) using the MaxQuant version 1.6.1.0 software (http://maxquant.org/). For samples analyzed using the data-independent analysis (DIA) approach, samples were processed using the suspension trap method (S-Trap, Protifi)^48^ where proteins are digested with a Trypsin/LysC mixture (Promega). LC-MS/MS was performed on a Orbitrap Eclipse (Thermo) in DIA mode: A scan cycle comprised of MS1 scan (*m*/*z* range from 390 to 1010, with a maximum ion injection time of 55 ms, a resolution of 60,000 and absolute automatic gain control (AGC) value of 4 × 10^5^) followed by 78 scan events of 8 *m*/*z* nonoverlapping windows. Fragmentation was carried out in HCD mode and Normalized collision energy was set to 33%. MS2 scans were carried out at a resolution of 15,000 with an absolute AGC value of 5.0e5. Data was searched using DIA-NN version 1.8.1 with *in silico* generation of a spectral library against the *P. falciparum* 3D7 proteome (Uniprot, accession 36329) and an in-house human erythrocyte proteome (Uniprot, accession 9606). Trypsin/P was set as a protease with one missed cleavage permitted, N-term M excision, oxidation (M), and acetylation (N-term) were included as variable modifications while carbamidomethylation (C) was included as fixed modification. Unrelated runs and MBR options were enabled, cross-run normalization was disabled, and all other settings were kept default. All pulldown proteomics data sets have been deposited in the ProteomeXchange Consortium via the PRIDE^43^ partner repository under the identifier **PXD075229.**

### Isothermal Thermal Proteome Profiling (iTPP) Lysate Preparation

*P. falciparum* lysates for iTPP were prepared as previously described.^28^ Briefly, late-trophozoite/schizont stage cultures at high parasitaemia (8–15%) were harvested by centrifugation (1800*g*, 15 min, brake 2, RT). Pellets were washed in incomplete malaria media (IMM; as complete media IMM, but without albumax II) then resuspended in 5 pellet volumes of IMM before late-trophozoite and schizont infected erythrocytes were isolated using a SuperMACS II magnet with a D-column. Magnet-enriched infected erythrocytes were lysed by incubation with 0.1% (v/v) saponin (10 min, on ice), then centrifuged (2800*g*, 8 min, 4 °C). The pellet was washed 3× in wash buffer (100 mM potassium acetate, 2.5 mM magnesium acetate, 45 mM pH 7.4 HEPES, 250 mM sucrose, 2 mM dithiothreitol [DTT], 15 μM leupeptin) with centrifugation (2800*g*, 8 min, 4 °C). Finally, extracellular parasites were lysed via nitrogen cavitation (Parr; 1500 psi, 60 min, on ice). The resultant lysate was cleared by centrifugation (100,000*g*, 20 min, 4 °C) and the protein content of the supernatant determined by Bradford assay.

### iTPP

Isothermal TPP was performed essentially as previously described.^28^ In this instance *P. falciparum* lysates incubated ± drug were incubated at 37 and 48 °C. Sample processing, fractionation, protein identification and quantitation was performed as described for chemical pulldown. All iTPP proteomics data sets have been deposited in the ProteomeXchange Consortium via the PRIDE^43^ partner repository under the identifier **PXD075163.**

### Data Analysis

Pulldown and iTPP data were visualized in Perseus version 1.6.15 (https://maxquant.net/perseus) and dose–response curves were generated in R studio using the *drc* package.

### *Pf*PK6 Recombinant Expression, Purification, and Enzymatic Assay

*Pf*PK6 recombinant expression, purification and enzymatic assessment via ADP-glo kinase activity and radiometric kinase assays were performed as previously described.^24^

### In-Gel Radioactive Assay

Kinase reactions to measure PfPK6 inhibition were performed using 0.5 μg of recombinant *Pf*PK6-GST and 2 μg of recombinant substrate, Histone H1 (Cayman Chemical), in a total volume of 20 μL. The kinase buffer (25 mM Tris-HCl pH 7.5, 5 mM ß-glycerophosphate, 2 mM DTT, 0.1 mM Na_3_VO_4_, 10 mM MgCl_2_) (Cell Signaling), supplemented with 2 mM MnCl_2_, 300 μM ATP, and 5 μCi (γ-^32^P) ATP. Inhibitors were screened using a 1:3 serial dilution starting at 10 μM. Staurosporine was used as a control at a concentration of 1 μM. The enzyme was preincubated with the inhibitors for 10 min before addition of the substrate and ATP. Kinase reactions were then incubated for 30 min at RT, after which 7 μL NuPAGE LDS sample buffer (Thermo Fisher) were added to each tube to terminate the reaction. Samples were boiled for 5 min, spun, and loaded onto a 12% SDS/Bis-Tris polyacrylamide gel containing 2,2,2-trichloroethanol (TCE). The gel was imaged by fluorescence visualization and transferred to a PVDF membrane using the Bio-Rad semidry transfer system, dried, and then placed into a phosphor-screen cassette for 4 h. Autoradiographs were captured using a BioRad storage phosphor-imager.

### Conditional Knockdown (cKD) of *P. falciparum* PK6

*Pf*PK6 (PF3D7_1337100) cKD lines were generated by fusing the coding sequence and noncoding RNA aptamer sequences in the 5′- and 3′-UTR, permitting translation regulation using the TetR-DOZI system.^30^ Editing was achieved by CRISPR/SpCas9 using the linear pSN054 vector that contains cloning sites for the left homology region (LHR) and the right homology region (RHR) as well a gene-specific guide RNA under control of the T7 promoter.^31^ The final constructs were verified by Sanger sequencing. Transfection into Cas9- and T7 RNA polymerase-expressing NF54 parasites was carried out by preloading erythrocytes with the donor vector as previously described.^49^ Parasite culture was maintained continuously in 500 nM anhydrotetracycline (aTc, Sigma-Aldrich) and drug selection with 2.5 μg/mL of Blasticidin S (RPI Corp) was initiated 4 days after transfection.

Compound susceptibility assays were carried out using *Pf*PK6 cKD lines maintained with aTc (500 nM) for wild-type expression or no aTc for target knockdown conditions. Cultures were distributed into 384-well polystyrene microplates (Corning), and a stock solution of MMV022224 was serially diluted and transferred to the parasite-containing plates using the Janus liquid handler (PerkinElmer). DMSO and dihydroartemisinin treatment (500 nM) served as reference controls. Luminescence was measured after 72 h using the Renilla-Glo Luciferase Assay System (Promega) and the GloMax Discover Multimode Microplate Reader (Promega), and IC_50_ values were obtained from corrected dose–response curves using Graph-Pad Prism.

### Western Blotting

To assess regulation of the PK6 protein expression, cKD parasites were cultured in the presence (500 nM) and absence of aTc. Protein samples were extracted after 72 h via saponin lysis and resuspended in parasite lysis buffer that consists of 4% SDS and 0.5% Triton X-114 in PBS. Proteins were separated on a Mini-PROTEAN TGX Precast Gels (4–15% gradient) in tris-glycine buffer, transferred to a polyvinylidene fluoride (PVDF) membrane using the Mini Trans-Blot Electrophoretic Transfer Cell system according to the manufacturer’s instructions, and blocked with 100 mg/mL skim milk in TBS/Tween. Membrane-bound proteins were probed with mouse anti-HA (1:3000; Sigma) and rabbit anti-GAPDH (1:5000; Abcam) primary antibodies, and antimouse (1:5000; Thermo Fisher Scientific) and antirabbit (1:5000; Cell Signaling) horseradish peroxidase (HRP)-conjugated secondary antibodies. Following incubation in SuperSignal West Pico Chemiluminescent substrate (Thermo Fisher Scientific), protein blots were imaged and analyzed using the ChemiDoc MP System and Image Lab 5.2.0 (Bio-Rad).

## Supplementary Material



## Data Availability

No bespoke code was used in this study. Proteomics data sets have been deposited in the ProteomeXchange Consortium via the PRIDE^43^ partner repository under the identifier **PXD075163** (iTPP) and **PXD075229** (chemical pulldown).

## Notes

Representation of specific transgenic barcoded cell lines within the AReBar library population prior to and following selection with MMV022224 and TCMDC-135051 (Table S1), top hits from standard and dose–response pulldown studies with MMV022224 probe 1 (Table S2), top hits from isothermal TPP with MMV022224 (Table S3), top hits from standard and dose–response pulldown studies with TCMDC-135051 probe (Table S4), top hits from isothermal TPP with TCMDC-135051 (Table S5), sequences used in *Pf*PK6 cKD cell line generation (Table S6), relative potency of MMV022224 and TCMDC-135051 against wild type Dd2 and CRISPR-edited parasites (Table S7), physicochemical and biological properties of MMV022224 (Table S8), cross resistance profiling of MMV022224 and TCMDC-135051 against 53 barcoded resistant parasite lines by AReBar assay (Figure S1), β-hematin formation inhibition assay data (Figure S2), in-gel radioactive assay showing inhibition of *Pf*PK6-mediated phosphorylation of histone H1 by MMV022224 and TCMDC-135051 (Figure S3), conditional knockdown of *Pf*PK6 (Figure S4), chemical pulldown with TCMDC-135051 probe 1 – dose response curves for CSK21 and CSK22 (Figure S5), *Pf*PK6 cKD aptamer regulatable line sequences (Figures S6 and S7) and complete chemical synthesis and characterization of final compounds and intermediates (PDF)

## #

Current address: Department of Infection Biology, Faculty of Infectious and Tropical Diseases, London School of Hygiene and Tropical Medicine, Keppel Street, London WC1E 7HT, United Kingdom (R.J.W.)

## &

Current address: Institute of Parasitology, Department of Infectious Diseases and Pathobiology, Vetsuisse Faculty, University of Bern, Länggassstrasse 122, 3012, Bern, Switzerland (N.W.).

## %

Current Address: LCC–CNRS, Laboratoire de Chimie de Coordination du CNRS UPR8241, Université de Toulouse, Inserm ERL 1289; 31077 Toulouse, France (F.N.).
