# Supplementary material for: Using Unbiased Chemical Proteomics Approaches to Explore the Target Landscape of the Resistance Refractory 7‑Azaindole MMV022224 in Plasmodium falciparum
Source: ACS Infect Dis. 2026 Jun 25;12(7):2332–44. doi: 10.1021/acsinfecdis.6c00276 (PMC13366577; doi:10.1021/acsinfecdis.6c00276)
Supplement: Supplementary file 1 [file id6c00276_si_001.pdf]

Supporting information

Using unbiased chemical proteomics approaches to explore the target landscape of the resistance refractory 7-azaindole MMV022224 in *Plasmodium falciparum*

Aisha J. Syed[a], Rachel Milne[a], Victoriano Corpas-Lopez[a], Gourav Dey[a], Nonlawat Boonyalai[b], Richard J. Wall[a,#], Natalie Wiedemar[a,&], Lukas Montejo[c], Flore Nardella[c, %], Kathryn J. Wicht[d], Charisse Flerida A. Pasaje[e], John Okombo[f,g], Jacquin C. Niles[e], David A. Fidock[f,g], Marcus C. S. Lee[b], Debopam Chakrabarti[c], Stephen Patterson[a,*] and Susan Wyllie[a,*]

[a] Drug Discovery Unit, Division of Drug Discovery, Faculty of Life Sciences, University of Dundee, Dow Street, Dundee DD1 5EH, United Kingdom

[b] Division of Biological Chemistry and Drug Discovery, Faculty of Life Sciences, University of Dundee, Dow Street, Dundee DD1 5EH, United Kingdom

[c] Division of Molecular Microbiology, Burnett School of Biomedical Sciences, University of Central Florida, 12722 Research Parkway, Orlando, Florida 32826, United States.

[d] Holistic Drug Discovery and Development (H3D) Centre, Department of Chemistry and Institute of Infectious Disease and Molecular Medicine, University of Cape Town, Rondebosch 7701, South Africa

[e] Department of Biological Engineering, Massachusetts Institute of Technology, Cambridge, MA 02139, United States

[f] Department of Microbiology & Immunology, Columbia University Irving Medical Center, New York, NY 10032, USA

[g] Center for Malaria Therapeutics and Antimicrobial Resistance, Division of Infectious Diseases, Department of Medicine, Columbia University Irving Medical Center, New York, NY 10032, USA

[*]Corresponding authors: Susan Wyllie (s.wyllie@dundee.ac.uk), Stephen Patterson (s.z.patterson@dundee.ac.uk)

[#]Current address: Department of Infection Biology, Faculty of Infectious and Tropical Diseases, London School of Hygiene and Tropical Medicine, Keppel Street, London WC1E 7HT, United Kingdom

[&]Current address: Institute of Parasitology, Department of Infectious Diseases and Pathobiology, Vetsuisse Faculty, University of Bern, Länggassstrasse 122, 3012, Bern, Switzerland

Table S1: Representation of specific transgenic barcoded cell lines within the AReBar library population prior to and following selection with MMV022224 and TCMDC-135051.

Cell line	Gene descriptor	Gene ID	Day 0 (%)	Day 14 (%)	Day 14 (%)	Day 14 (%)
			Untreated	Untreated	MMV022224	TCMDC-135051
3D7	wild type	-	9.64	17.81	3.98	1.94
3D7 ABCI3 R2180P	ABC transporter I family member 1	PF3D7_0319700	5.75	7.28	3.35	1.12
3D7 ACS10 M300I	Acyl CoA synthase	PF3D7_0525100	4.37	2.50	3.25	0.89
3D7 ACS11 D648Y	Acyl CoA synthase	PF3D7_1238800	1.38	0.24	0.69	0.39
3D7 ATP2 CNV2	Phospholipid-transporting ATP2	PF3D7_1219600	6.32	7.17	3.66	1.33
3D7 DHFR-TS G378E	Dihydrofolate reductase-thymidylate synthase	PF3D7_0417200	4.68	9.78	2.44	1.36
3D7 DHFR-TS I403L	Dihydrofolate reductase-thymidylate synthase	PF3D7_0417200	5.29	7.79	2.77	1.21
3D7 FTb A515T	Farnesyltransferase subunit beta	PF3D7_1147500	6.27	7.04	3.69	1.31
3D7 MDR2 K840N	Multidrug resistance protein 2	PF3D7_1447900	5.02	4.36	2.42	0.96
3D7 NCR1 A1108T	Niemann-Pick type C1-related protein	PF3D7_0107500	5.95	7.35	2.92	1.29
Dd2	wild-type	-	3.08	2.24	4.66	0.70
Dd2 AcAS A597V	Acetyl-CoA synthetase	PF3D7_0627800	0.49	0.05	1.30	0.10
Dd2 AcAS T648M	Acetyl-CoA synthetase	PF3D7_0627800	1.02	1.39	2.31	0.19
Dd2 AsnRS R487S	Asn-tRNA synthetase	PF3D7_0509600	1.07	0.43	1.47	0.33
Dd2 ATP4 G358S	Non-SERCA-type Ca2+ -transporting P-ATPase	PF3D7_1211900	0.78	0.43	1.23	0.18
Dd2 ATP4 L927V	Non-SERCA-type Ca2+ -transporting P-ATPase	PF3D7_1211900	0.99	0.21	2.04	0.28
Dd2 ATP4 Q172H	Non-SERCA-type Ca2+ -transporting P-ATPase	PF3D7_1211900	0.63	0.36	0.92	0.17
Dd2 CARL I1139K-a	Cyclic amine resistance locus	PF3D7_0321900	0.00	0.00	0.01	0.00
Dd2 CARL I1139K-b	Cyclic amine resistance locus	PF3D7_0321900	0.17	0.09	0.26	0.04
Dd2 CARL V1103L	Cyclic amine resistance locus	PF3D7_0321900	0.46	0.20	0.90	0.09

Dd2 CLK3 H259P	Cyclin-dependent-like kinase	PF3D7_1114700	0.89	0.73	2.10	78.82
Dd2 CPSF Y408S E	Cleavage and polyadenylation specific factor	PF3D7_1438500	0.63	0.14	0.57	0.16
Dd2 CPSF Y408S S	Cleavage and polyadenylation specific factor	PF3D7_1438500	0.50	0.00	0.26	0.18
Dd2 CRT M343L	Chloroquine resistance transporter	PF3D7_0709000	2.00	0.37	2.46	0.37
Dd2 CSC1 L800P	CSC1-like protein, putative	PF3D7_1250200	0.67	0.08	0.91	0.15
Dd2 cytBC1 G33V	Cytochrome b	PF3D7_MIT02300	1.58	0.03	2.53	0.30
Dd2 cytBC1 V284L	Cytochrome b	PF3D7_MIT02300	0.60	0.14	1.24	0.11
Dd2 DHFR-TS S216R	Dihydrofolate reductase-thymidylate synthase	PF3D7_0417200	1.25	0.23	1.99	0.27
Dd2 DHODH C276Y	Dihydroorotate dehydrogenase	PF3D7_0603300	0.76	0.14	0.89	0.17
Dd2 DHODH F227I	Dihydroorotate dehydrogenase	PF3D7_0603300	0.36	0.00	0.32	0.09
Dd2 DHODH I263F	Dihydroorotate dehydrogenase	PF3D7_0603300	0.07	0.00	0.21	0.02
Dd2 DHODH L531F	Dihydroorotate dehydrogenase	PF3D7_0603300	0.38	0.01	0.51	0.05
Dd2 eEF2 L755F	Elongation factor 2	PF3D7_1451100	0.56	0.16	1.34	0.13
Dd2 eEF2 Y186N	Elongation factor 2	PF3D7_1451100	0.47	0.02	0.52	0.10
Dd2 GGPPS S228T	Geranylgeranyl diphosphate synthase	PF3D7_1128400	0.68	0.18	1.64	0.14
Dd2 HSP90 A41S	Heat shock protein 90	PF3D7_0708400	1.64	2.43	3.33	0.27
Dd2 IleRS E180D	Ile-tRNA synthetase	PF3D7_1332900	1.31	0.40	2.17	0.28
Dd2 IleRS L810F	Ile-tRNA synthetase	PF3D7_1332900	1.15	0.52	2.43	0.22
Dd2 IleRS V500A	Ile-tRNA synthetase	PF3D7_1332900	1.13	0.37	1.63	0.28
Dd2 kelch13 C580C	Kelch protein K13	PF3D7_1343700	1.59	0.45	2.08	0.31
Dd2 kelch13 C580Y	Kelch protein K13	PF3D7_1343700	1.14	0.44	2.26	0.20
Dd2 kelch13 R539T	Kelch protein K13	PF3D7_1343700	0.51	0.07	0.65	0.19
Dd2 MCP D195N	Mitochondrial carrier protein	PF3D7_0908800	0.24	0.01	0.59	0.03
Dd2 MCP P214T	Mitochondrial carrier protein	PF3D7_0908800	1.33	6.70	1.74	0.27
Dd2 PI4K S1320L+L1418F	Phosphatidylinositol 4-kinase	PF3D7_0509800	1.28	0.18	1.57	0.33
Dd2 PI4K S743F+H1484Y	Phosphatidylinositol 4-kinase	PF3D7_0509800	0.85	0.34	1.50	0.14

Dd2 ProRS L482H	Pro-tRNA synthetase	PF3D7_1213800	2.82	0.06	2.82	0.55
Dd2 PROTB5 A20V	Proteasome β5 26S (A80V immature)	PF3D7_1011400	1.01	0.32	1.30	0.22
Dd2 PROTB5 M45I	Proteasome β5 26S (M105I immature)	PF3D7_1011400	0.83	0.74	1.32	0.17
Dd2 QRP1 D1863Y	Quinoxaline resistance protein	PF3D7_1359900	1.97	4.13	2.91	0.40
Dd2 TyrRS S234C	Tyr-tRNA synthetase	PF3D7_1117500	1.58	0.84	2.48	0.57
Dd2 UDP-GT F37V	UDP-galactose transporter	PF3D7_1113300	0.35	0.01	0.28	0.13
Dd2 yDHODH	Yeast dihydroorotate dehydrogenase	-	4.48	3.03	7.17	0.51

Table S2: Top hits from standard and dose-response pulldown studies with MMV022224 probe 1.

Log_2 enrichment DMSO / MMV022224	Dose response IC_{50} (± SD), µM	Gene ID	Name
5.02	0.16 ± 0.03	PF3D7_1337100.1	protein kinase 6 (*Pf*PK6)
2.52	1.04 ± 0.8	PF3D7_0311300.1	phosphatidylinositol 3- and 4-kinase, putative (*Pf*PI(3,4)K)
2.15	1.2 ± 2.6	PF3D7_1114700.1	cyclin-dependent-like kinase (*Pf*CLK3)
1.88	2.6 ± 0.06	PF3D7_0934800.1	cAMP-dependent protein kinase catalytic subunit
1.80	6.5 ± 26	PF3D7_0810200.1	ABC1 family, putative
1.77	NA	S100A8	Human protein S100-A8
1.64	NA	SERPINB4	Human serpin B4
1.63	NA	IQGAP2	Human IQ motif containing GTPase activating protein 2
1.51	2.1 ± 0.8	PF3D7_1223100.1; PF3D7_1444500.1	cAMP-dependent protein kinase regulatory subunit
1.45	0.08 ± 0.18	PF3D7_0532300.1	*Plasmodium* exported protein (PHISTb), unknown function
1.40	0.005 ± 0.005	EEF1A1P5	Human putative elongation factor 1-alpha-like 3
1.37	16 ± 67	PF3D7_1105100.1	histone H2B
1.34	0.2 ± 100	PF3D7_0804600.1	tRNA pseudouridine synthase, putative
1.25	3.3 ± 10	PF3D7_1332200.1	conserved protein, unknown function
1.04	NA	EIF2B5	Human eukaryotic translation initiation factor 2B subunit epsilon
1.03	26 ± 43	PF3D7_1136500.1; PF3D7_1136500.2	casein kinase 1
1.00	7.2 ± 21	SELENOO	Human protein adenylyltransferase SelO, mitochondrial

0.98	11 ± 4	PF3D7_0505500.1	DNA mismatch repair protein MSH6, putative
0.97	8 ± 15	SKP1	Human S-phase kinase-associated protein 1
0.95	NA	STAMBP	Human STAM-binding protein

Table S3: Top hits from isothermal TPP with MMV022224.

Log$_2$ change in abundance MMV022224 / DMSO Rep 1	Log$_2$ change in abundance MMV022224 / DMSO Rep 2	Gene ID	Name
2.07	4.17	PF3D7_1337100.1	protein kinase 6 (*Pf*PK6)
0.24	5.07	PF3D7_0106300.1	calcium-transporting ATPase
0.84	1.37	PF3D7_1114700.1	cyclin-dependent-like kinase (*Pf*CLK3)
-0.57	2.73	PF3D7_1230500.1	WD repeat-containing protein, putative
0.66	1.39	PF3D7_0812100.1	Proteasome activator complex subunit 4, putative
0.38	1.65	PF3D7_1306900.1	U1 small nuclear ribonucleoprotein A, putative
0.26	1.69	PF3D7_1126200.1	40S ribosomal protein S18, putative
0.22	1.72	PF3D7_0405400.1	pre-mRNA-processing-splicing factor 8, putative
0.36	1.39	PF3D7_0520000.1	40S ribosomal protein S9, putative
0.79	0.95	PF3D7_0509600.1	asparagine-tRNA ligase
-0.08	1.80	PF3D7_1242000.1	Sulfhydryl oxidase
0.48	1.20	PF3D7_0519200.1	V-type proton ATPase proteolipid subunit
0.52	0.98	PF3D7_0903400.1	ATP-dependent RNA helicase DDX60, putative
0.35	1.14	PF3D7_1205100.1	O-phosphoseryl-tRNA(Sec) selenium transferase
0.03	1.46	PF3D7_1239700.1	ATP-dependent zinc metalloprotease FTSH 1
0.39	1.10	PF3D7_1315400.1	zinc finger (CCCH type) protein, putative
0.21	1.24	PF3D7_0522400.1	Chorein *N*-terminal domain-containing protein
0.24	1.17	PF3D7_1121100.1	rhoptry protein
0.87	0.51	PF3D7_1142700.1	methyltransferase, putative
0.30	1.07	PF3D7_0516200.1	40S ribosomal protein S11

Table S4: Top hits from standard and dose-response pulldown studies with TCMDC-135051 probe. Primary targets highlighted in grey. Enriched casein kinases (human and parasite) are highlighted in pink.

Log_2 enrichment DMSO / TCMDC-135051	Dose response IC_{50} (± SD), μM	Gene ID	Name
5.87	0.001 ± 0.0011	PF3D7_1114700.1	cyclin-dependent-like kinase 3 (*Pf*CLK3)
5.16	1.08 ± 0.6	PF3D7_1337100.1	protein kinase 6 (*Pf*PK6)
4.65	1.2 ± 1.5	PF3D7_1366500.1	nucleoside diphosphate kinase
3.80	0.95 ± 0.3	PF3D7_0727700.1	conserved *Plasmodium* protein, unknown function
3.17	0.0009 ± 0.004	CSNK2A2	Human casein kinase II subunit alpha OS
2.39	NA	PF3D7_0723400.1	conserved *Plasmodium* protein, unknown function
2.37	0.86 ± 1.4	PF3D7_1108400.1	casein kinase 2, alpha subunit
2.04	NA	PF3D7_0510200.1	peptidyl-prolyl cis-trans isomerase
2.01	1.2 ± 86	PF3D7_1342400.1	casein kinase II beta chain
1.81	NA	PF3D7_0205400.1	PCI domain-containing protein, putative
1.80	NA	PF3D7_1114500.1	conserved *Plasmodium* protein, unknown function
1.77	0.0014 ± 0.005	CSNK2A1	Human casein kinase II subunit alpha OS
1.70	NA	PF3D7_0926100.1	protein kinase, putative

1.64	NA	PF3D7_1005600.1	DnaJ protein, putative
1.58	NA	PF3D7_0927700.1	serine/threonine protein phosphatase 4, putative
1.47	NA	PF3D7_0504000.1	cation transporting P-ATPase
1.46	0.97 ± 0.65	PF3D7_0317700.1	CPSF (cleavage and polyadenylation specific factor), subunit A, putative
1.41	1.7 ± 4.7	PF3D7_1103700.1	casein kinase II beta chain
1.36	114 ± 5619	PF3D7_0617800.1	histone H2A
1.32	NA	PF3D7_1438900.1	thioredoxin peroxidase 1

Table S5: Top hits from isothermal TPP with TCMDC-135051.

Log_2 change in abundance TCMDC-135051 / DMSO Rep 1	Log_2 change in abundance TCMDC-135051 / DMSO Rep 2	Gene ID	Name
3.60	2.90	PF3D7_1114700.1	cyclin-dependent-like kinase (*Pf*CLK3)
1.18	0.97	PF3D7_1337100.1	protein kinase 6 (*Pf*PK6)
1.22	0.07	PF3D7_0627700.1	Transportin
0.66	0.50	PF3D7_1436600.1	cGMP-dependent protein kinase
0.56	0.57	PF3D7_1456700.1	Armadillo repeat protein
0.82	0.30	PF3D7_0728900.1	RNA-binding protein, putative
0.53	0.55	DCD	Human Dermcidin
0.28	0.78	PF3D7_1467300.1	1-deoxy-D-xylulose 5-phosphate reductoisomerase, apicoplastic
0.17	0.81	PF3D7_1021900.1	PHAX domain-containing protein, putative
-0.03	1.00	PF3D7_1033900.1	E2 ubiquitin-conjugating enzyme
0.77	0.15	EEF2	Human elongation factor 2
0.80	0.12	TGM2	Human protein-glutamine gamma-glutamyltransferase 2
1.04	-0.13	PF3D7_1116500.1	Folate transporter 2
0.81	0.10	PF3D7_0910100.1	Exportin-7, putative
0.16	0.71	PF3D7_0520800.1	Uncharacterized protein
0.54	0.31	PF3D7_0817700.1	Rhoptry neck protein 5
0.51	0.30	PF3D7_1401800.1	Choline kinase
0.02	0.78	PF3D7_0908600.1	Putative tRNA (cytidine(32)/guanosine(34)-2'-O)-methyltransferase
0.31	0.48	RAD23B	Human UV excision repair protein RAD23 homolog B
0.81	-0.03	PF3D7_0617900.1	Histone H3

Table S6: Sequences used in *Pf*PK6 cKD cell line generation. List of oligonucleotides for donor vector construction. LHR and RHR signifies left and right homology regions, respectively.

Sequence description	Nucleotide sequence (5´ - 3´)
LHR reverse	attggttttcaaacttcattgactgtgccggccggccTATACTGACTTTTGTTGTATATCTTAT
LHR reverse	cacattccgtccgtatcgacctttctctgcctggatTTGGACATTATATAAATTTTTTATTTTAA
RHR reverse	gtacggtacaaacccggaattcgagctcggGAAAAAAAAGATAGAGAACATAAG
RHR reverse	gggtattagacctagggataacagggtaatGAGCAAAAACGGAAGGTAAA
sgRNA target site	TGCTCAATGTCCTGGAAACA

Table S7: Relative potency of MMV022224 and TCMDC-135051 against wild type Dd2 and CRISPR-edited parasites.

Cell line	MMV022224		TCMDC-135051	
	EC_{50} value ± SD, µM	Fold-shift relative to Dd2 parental	EC_{50} value ± SD, µM	Fold-shift relative to Dd2 parental
**Dd2**	0.3 ± 0.008	-	0.2 ± 0.007	-
***Pf*CLK3^{H259P}**	0.4 ± 0.009	1.2	1.2 ± 0.1	5

EC_{50} values represent the mean ± SD of at least 3 biological replicates

Table S8: Physicochemical and biological properties of MMV022224.[a]

P. falciparum EC_{50}	0.217 µM
HepG2 EC_{50}	3.39 µM
Selectivity index (HepG2 EC_{50}/*Pf* EC_{50})	15.6
Mouse Cl_{int}	5.76 mL min^{-1} g^{-1}
MDCK P_{app}	7.4 nm s^{-1}
Aqueous solubility pH7.4	205 µM (225 µM for di HCl salt)
Molecular weight	384.25
TPSA	35.16
cLogP / cLogD	3.93 / 2.7

[a]Assays performed as described [1].

Supplemental figures

Figure S1: Cross resistance profiling of MMV022224 and TCMDC-135051 against 53 barcoded resistant parasite lines by AReBar assay. (A) Growth of pool treated either with MMV022224, TCMDC-135051, the positive control DHODH inhibitor DSM265, or no-drug treatment. Data are mean ± SD, triplicate values. (B) Barcode profiles showing proportion of each line at day 0 (input) and day 14 for the pool treated with either MMV022224, TCMDC-135051, DSM265, or the no-drug control.

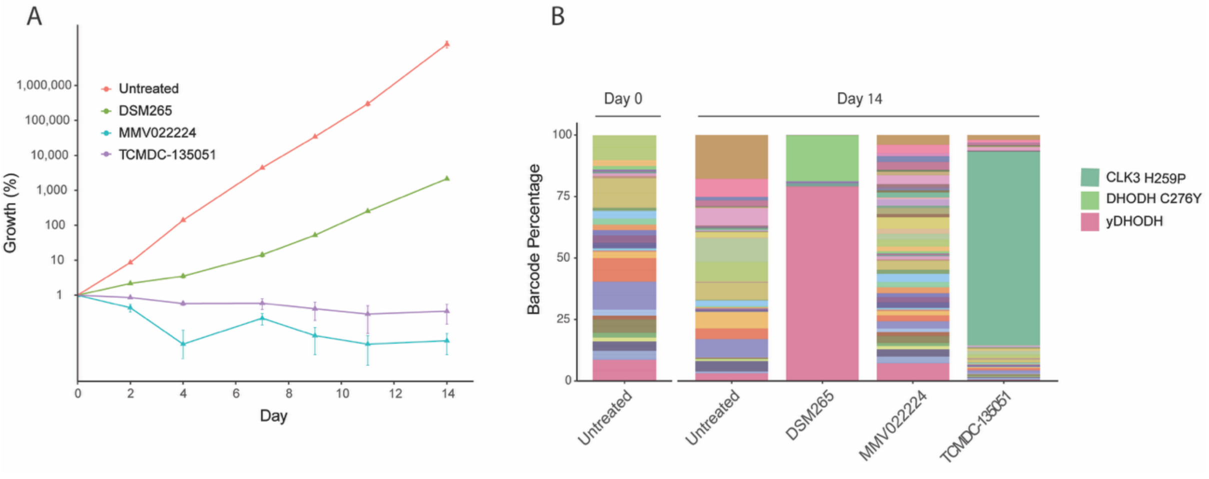

Figure S2: β-hematin formation inhibition assay. (**A**) Dose response curves for MMV022224 (red) and chloroquine (positive control, black). (B) IC_{50} values for inhibition of β-hematin formation. Values present the mean ± SD of triplicate measurements.

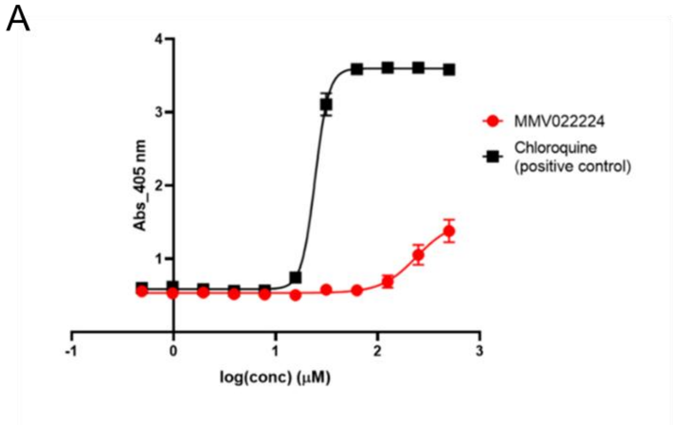

B

Test compound	Inhibition of β-haematin formation, IC_{50} ± SD, µM*
Chloroquine (positive control)	24 ± 1
MMV022224	>200

Figure S3: In-gel radioactive assay showing inhibition of *Pf*PK6-mediated phosphorylation of histone H1 by MMV022224 and TCMDC-135051. Top: autoradiograph; bottom: trichloroethanol (TCE) in-gel visualization. Assay: samples containing 100 µM ATP, 5 µCi-γ32P ATP (3000 Ci/mmol), 0.5 µg *Pf*PK6-His, histone H1 and increasing concentrations of test compounds were resolved on a 12% Bis-Tris gel impregnated with TCE. TCE-gel was visualized by UV in a transilluminator, transferred onto a PVDF membrane, dried and the radioactive signal was detected using a phosphorimager. Staurosporine at 1 µM was used as a control. The picture is representative of 3 independent experiments.

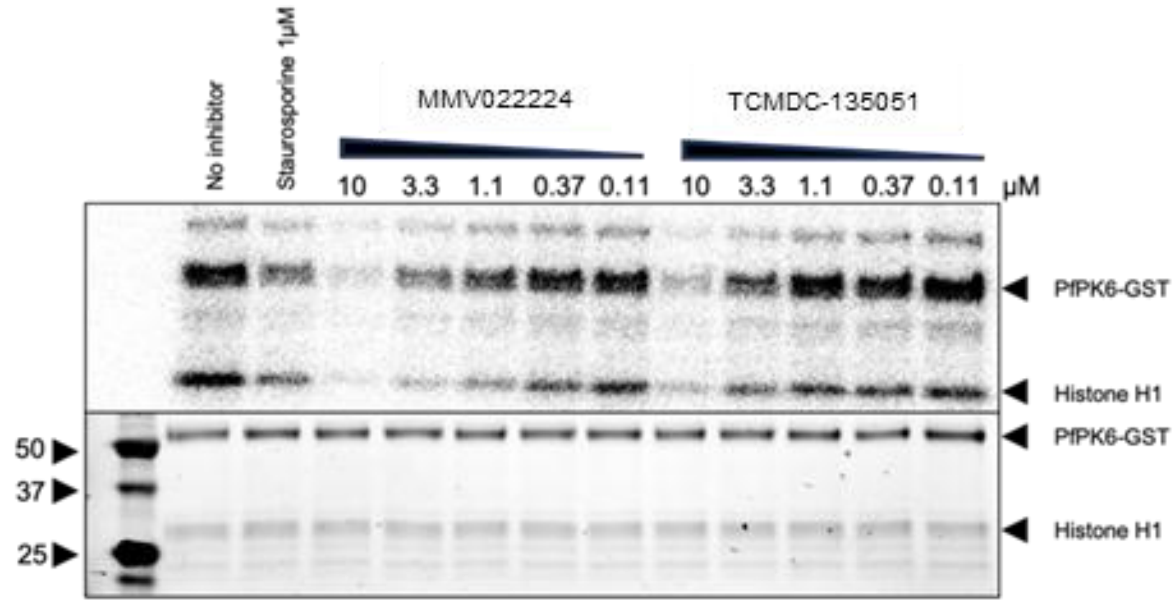

Figure S4: Conditional knockdown of *Pf*PK6. Schematic representation of modest (**A**, 10× aptamers added to the 3´ end of the gene of interest (GOI)) and strong (**B**, 10× aptamers added to both the 5´ and 3´ends of the GOI) conditional knockdown strategies. **C**) Growth profile of parasites following induction of modest *Pf*PK6 knock-down. **D**) Western blot confirming knockdown (modest) of HA-tagged *Pf*PK6 following removal of aTc for 72 h. Glyceraldehyde-3-phosphate dehydrogenase (GAPDH) used as a loading control. **E**) Growth profile of parasites following induction of strong *Pf*PK6 knock-down. **F**) Western blot confirming knockdown (strong) of HA-tagged *Pf*PK6 following removal of aTc for 72 h. GAPDH used as a loading control. MMV022224 dose response curves for conditional knockdown cell lines the absence (red) and presence (blue) of aTc. Knockdown under modest and strong conditions shown in **G** and **H**, respectively. EC_{50} values of 118 and 113 nM were observed in the absence (modest knock down conditions, red) and presence of aTc (control, blue), respectively. EC_{50} values of 250 and 190 nM were observed in the absence (stringent knockdown conditions, red) and presence (control, blue) of aTc, respectively.

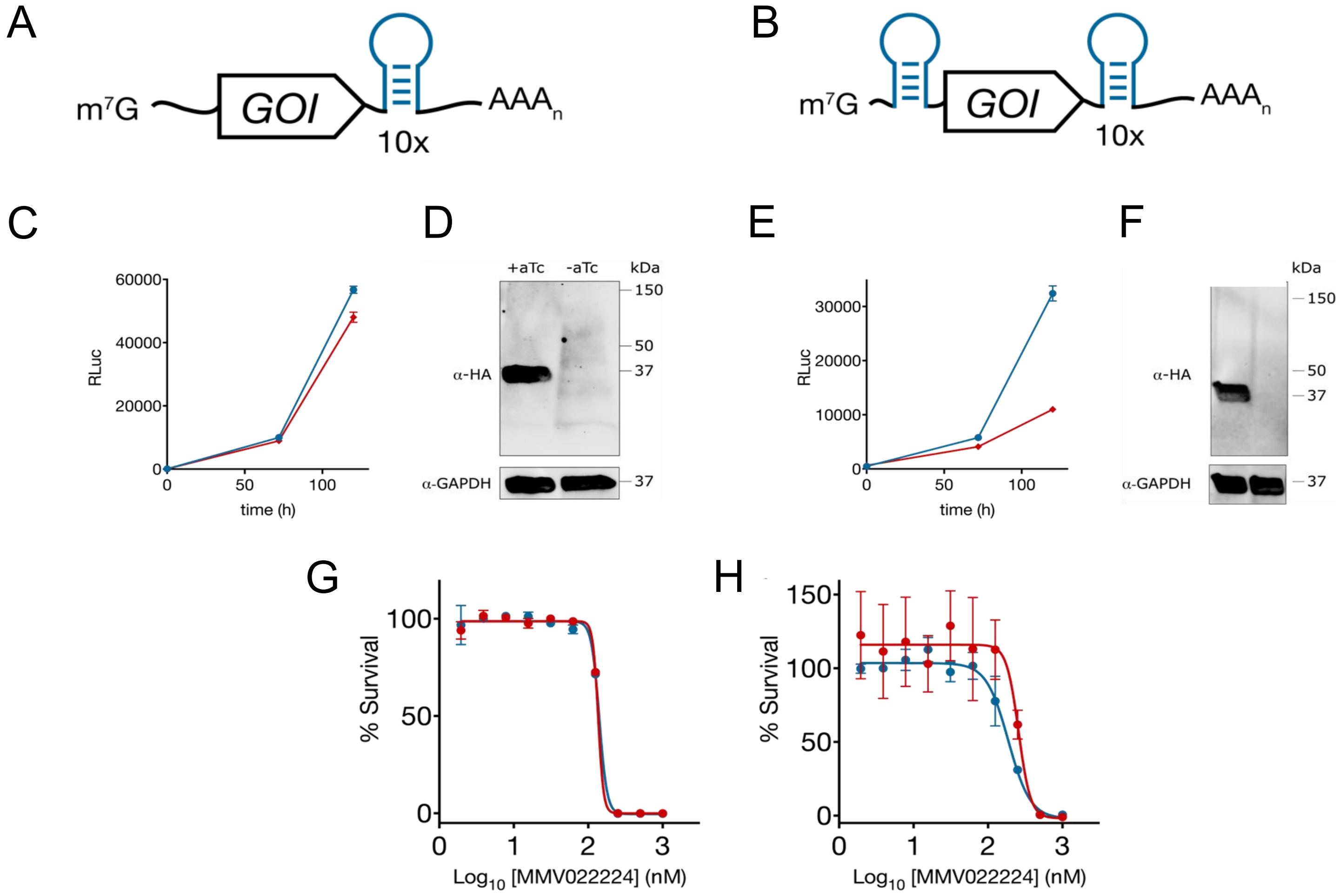

Figure S5: Chemical pulldown with TCMDC-135051 probe 1. Relative abundance of CSK21 and 22 in the presence of dose dependent competition with TCMDC-135051. IC_{50} values of 0.0009 ± 0.004 and 0.0014 ± 0.005 μM were established for CSK22 and CSK21, respectively.

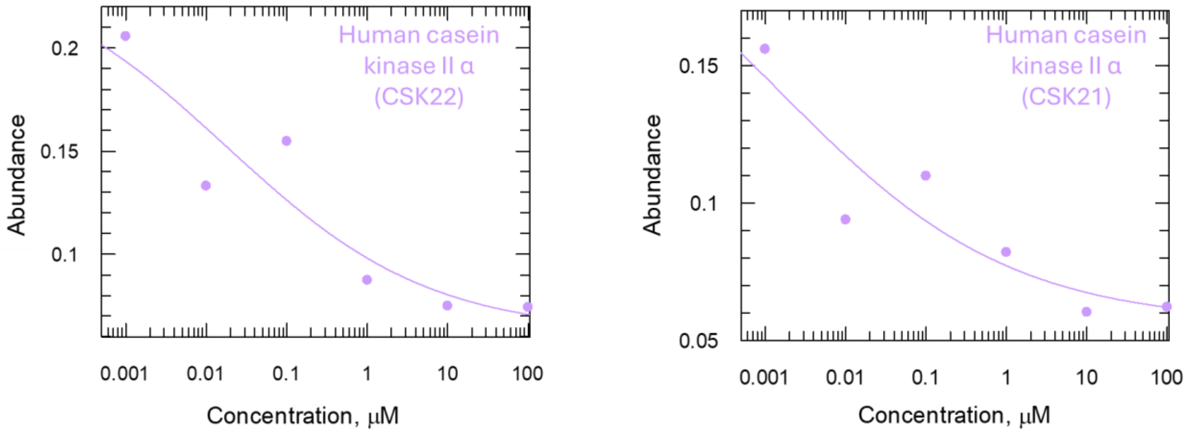

Figure S6: *Pf*PK6 3′-aptamer regulatable cKD line sequence. Left homology region (LHR) and re-codonized region (1202-1787 bp) shown. Stop codon has been removed.

CACTCAGgtataatccaaatgggcaatatatatatatatatatatatatatatatatatatatatattttatgtgtataatataaag
ttgtatatattttaaaaatatgtatatatgtaatatatatttatatatttatatatgtcagCTAGAACTGATTTATTTATTACTT
GGTGATAAGGATAAATTAACAACTGTTGACAAAGAAAGAAAGGATATGTTTCCATATTTTG
AGgtacgtagaaatatttcgagaaataaaaaaaatataaaatgaaaagataaaatataaaaactcaaattgatataatattaatt
aaaaaatatatatatatatatatttatatgtgtgtaattaaataaaaatctataccttattgtgaatacagatatttattacttcctgacctttt
attttttttttagATAAATATGTTAAAAGATGCTATAGATGATGAACATACATTAGATTTAATTTCTAAA
ATGTTAATATATGACCCTAACTACAGAATATCATCAAAAGAAGCTCTTAAACACCCATGCTT
TCAAGATATAGAACAGGTTAAGTTTAGCTACAACTTC

Figure S7: *Pf*PK6 dual 5′- and 3′-aptamer regulatable cKD sequence. Stop codon has been removed.

tatactgacttttgttgtatatcttatattttcttttttttttttttctccttcacattttaaattctcatatagtactgcagttattaataccaggaaaa
tatatataataaaataatcacgtatatattgaaaacaagaacaataaaaataaatttaaaaagaattaaacaaaattgaaaaaa
aaattgtgtataaaatattataatatataatatattatttgtataaaaaactaaattcaaaataaattttttttttttttttttaaattcaccca
agtcctataacaaggtaaggaaaagttcaaaataaaatatatctatatctatatatatatatgtaatattaatttatgtatatatatatat
atatatatatataataaaatataaaaattataattattgtaaaaaacttcatttactaattttatttatttattattattatttagttatttattttatc
ttattatttatgaatttatttttgttatcctaataaaaatatatattatatttataaggaaaatggttcttggatatattttaaaataaaaaattta
tataatgtccaaatccaggcagagaaaggtcgatacggacggaatgtgatggcctggataaaacacaaaactcgagaacatc
tgctaagcggtagtggtgaaggtagaggatcattattaacatgtggtgatgttgaagaaaatccaggtccacgtacgATGAAC
AGGATCGATATCAGCAACTTCGACTTCCTCTATGTGATCGGCAAAGGAACCTACGGAATC
GTGTACAAGGCTCTGGACAAGAAGGAGAACAACTTCGTGGCCATCAAGAAGATCATCAA
TCTGTGTGATGAGAACTATGGTATCAGCAAGTGCATCCTGCGCGAACTGACCATCCTGC
AGAAGATTAAGCACAAAAACATTATCAACCTGAAGTACGTGTTTTATGGCAAGGACATCG
AGGACAAGCTGAAGGGGGAAAACCTGGAGAATTCTTGCCTCTATCTGGCCTTCGAGTAC
TGTGACATCGATCTGTTTAACTTGATCAAGAAGCACAATCTGAACATCAAGGAGATTAAGT
ACATCATCTTCGAGCTGCTGCTGGCCCTCAGCTACTTCCATAGCAACAACTATATCCATC
GGGATATCAAGCCAGAGAACATCTTTATCACAAGCGAGGGCGAGATCAAGCTGGGCGA

CCTGGGCATGTCTGTGGAGAAGAGTGACCATATGACCCCGACGGTGGTGACCCTGTGG
TACCGGGCCCCTGAGATCCTGCTGAAGTCCACTAACTACGACCAGAAGGTGGATATCTG
GAGCCTGGGCTGCCTGTTTATGGAGCTGATTCAGGGCAGACCACTGTTCCCTGGCAAG
AACGACTGCACTCAGCTGGAGCTGATCTACCTGCTCCTGGGCGATAAAGACAAGCTGA
CCACAGTGGATAAGGAACGGAAGGACATGTTCCCTTACTTTGAGATCAACATGCTGAAG
GATGCCATCGACGATGAACACACCCTGGACCTGATCTCCAAGATGCTCATCTACGACCC
CAACTACAGAATTAGCAGCAAGGAGGCCCTGAAGCACCCCTGTTTCCAGGACATCGAAC
AGGTGAAGTTCAGCTACAACTTC

Chemical synthesis

Chemistry general

Solvents and reagents were purchased from commercial suppliers and used without further purification. Anhydrous solvents were purchased in Sure Seal™ bottles stored over molecular sieves. Air and moisture sensitive reactions were carried out under an inert atmosphere of nitrogen. Normal phase analytical thin-layer chromatography (TLC) was carried out on pre-coated silica plates (Kieselgel 60 F_{254}, BDH) with visualisation using UV light (UV 254/365 nm). Flash column chromatography was performed on a Combiflash Companion Rf (Teledyne ISCO) using prepacked silica gel columns (230-400 mesh, 35-70 µm, Teledyne ISCO). Reversed phase purifications were performed on the Teledyne ISCO ACCQPrep HP150 using a XBridge Prep C_{18} 5 µm OBO, 19 mm x 100 mm column eluted with 10 → 100% MeCN/0.1% NH_4OH (aq). ^{1}H NMR spectra were recorded on a Bruker Avance DPX 500 spectrometer (^{1}H at 500.1 MHz and ^{13}C at 125 MHz). Chemical shifts (δ) are expressed in ppm recorded using the residual solvent as the internal reference in all cases. Signal splitting patterns are described as singlet (s), doublet (d), triplet (t), quartet (q), multiplet (m), broad (br), or a combination thereof. Coupling constants (J) are quoted to the nearest 0.1 Hz. Low resolution electrospray (ES) mass spectra were recorded on an Advion Expression Compact Mass Spectrometer connected to a ThermoDionex Ultimate 3000 UPLC. High resolution mass spectra were recorded on either a Bruker MicrOTOF II focus ESI Mass Spectrometer connected in parallel to Dionex Ultimate 3000 RSLC system with diode array detector or an Orbitrap Exploris 120 Mass spectrometer. Compounds have been named using the ChemDraw Ultra 22.0 naming application which is commercially available from Revvity Signals Software. All stock solutions used in the synthesis of drug beads were prepared in high grade DMSO (Acros, 99.8%, for molecular biology). Drug bead reactions were mixed using a Miltenyi Biotec MACSmix Tube Rotator.

Synthetic procedures

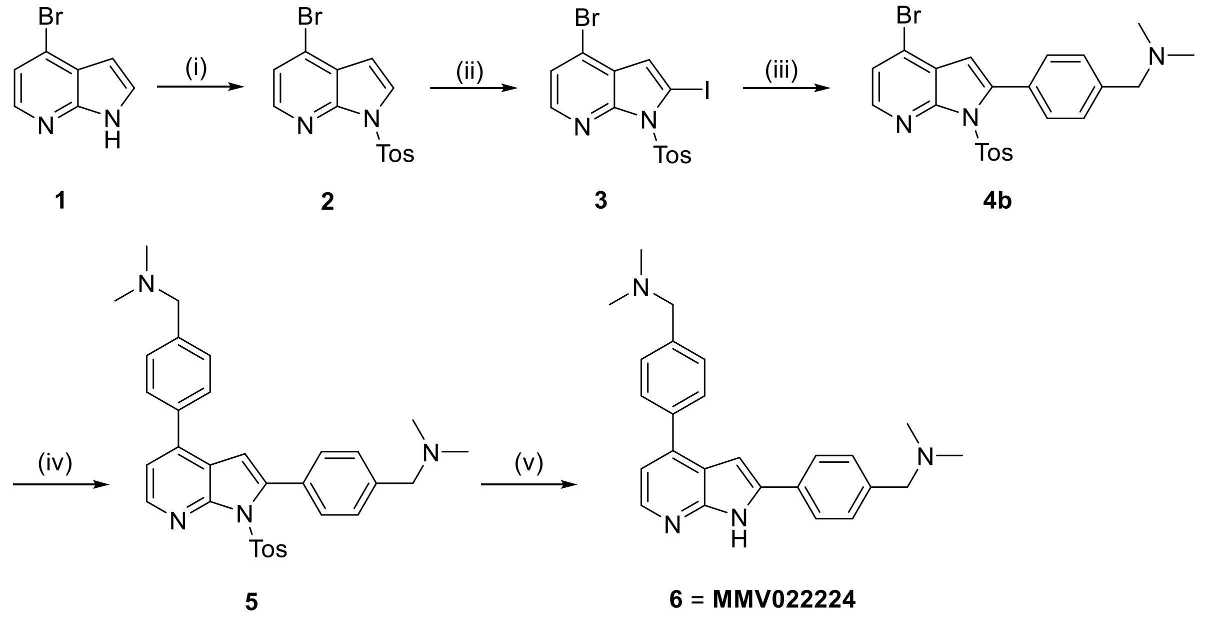

Scheme S1. Synthesis of MMV022224 (**6**).

Reagents and conditions: (i) NaH, TBAC, TosCl, THF, rt, 1 h, 87%; (ii) TEMED, LDA, I_2, THF, -78°C-rt, 17 h, 77%; (iii) *N*,*N*-dimethyl-1-[4-3(4,4,5,5-tetramethyl-1,3,2-dioxaborolan-2-yl)phenyl]methanamine, $Pd(PPh_3)_4$, K_3PO_4, 1,4-dioxane, 110°C, 16 h, 59%; (iv) *N*,*N*-dimethyl-

1-[4-(4,4,5,5-tetramethyl-1,3,2-dioxaborolan-2-yl)phenyl]methanamine, Pd(dppf)Cl_2, Na_2CO_3, 1,4-dioxane, 110°C, 16 h, 44%; (v) K_2CO_3, MeOH, 55°C, 16 h, 48%.

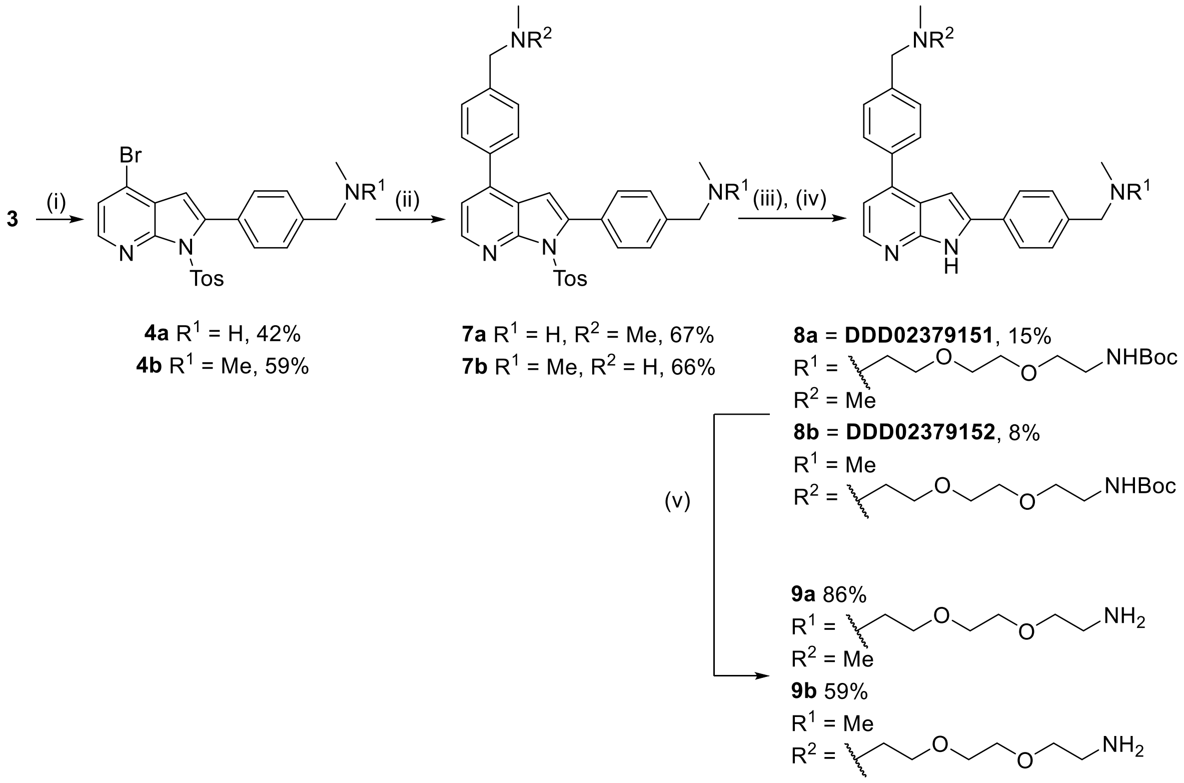

Scheme S2. Synthesis of MMV022224-based probes.

Reagents and conditions: (i) B(pin) ester, $Pd(PPh_3)_4$, K_3PO_4, 1,4-dioxane, 110°C, 16 h; (ii) B(pin) ester, Pd(dppf)Cl_2, Na_2CO_3, 1,4-dioxane, 110°C, 16 h; (iii) 2,2-dimethyl-4-oxo-3,8,11-trioxa-5-azatridecan-13-yl 4-methylbenzenesulfonate, Na_2CO_3, EtOH, 78°C, 16 h; (iv) K_2CO_3, MeOH, 65°C, 16 h; (v) For **8a** to **9a**: TFA, CH_2Cl_2, rt, 2 h, 86%; For **8b** to **9b**: HCl, dioxane, rt, 16 h, 59%.

2,2-dimethyl-4-oxo-3,8,11-trioxa-5-azatridecan-13-yl 4-methylbenzenesulfonate

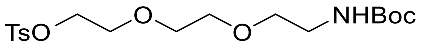

Prepared from *tert*-butyl (2-(2-(2-hydroxyethoxy)ethoxy)ethyl)carbamate using a literature method [2].

4-Bromo-1-tosyl-1*H*-pyrrolo[2,3-*b*]pyridine (2)

A solution of 4-bromo-1*H*-pyrrolo[2,3-*b*]pyridine (**1**) (1.00 g, 5.08 mmol) and 4-methylbenzenesulfonyl chloride (1.16 g, 6.09 mmol) in THF (16 mL) was added to a cooled suspension of NaH (60% in mineral oil) (609 mg, 15.2 mmol) at 0ºC and stirred for 15 min at 0ºC. A solution of tetrabutylammonium chloride (42.3 mg, 0.152 mmol) in THF (4 mL) was then added slowly to the resultant suspension over 5 min. The mixture was then allowed to warm to rt and further stirred for 1 h. The reaction was subsequently cooled to 0ºC and

quenched by the addition of water (10 mL). The aqueous layer was then separated and extracted with CH_2Cl_2 (3×20 mL). The combined organic layers were washed with brine (30 mL), dried over $MgSO_4$, filtered and concentrated under reduced pressure to yield the crude product as a brown oil, which was purified by flash chromatography (24 g silica, 0:100 → 20:80 EtOAc:heptane), to obtain the title compound as a white solid (1.56 g, 4.44 mmol, 87%). ^1H NMR (500 MHz, $CDCl_3$) δ 8.24 (1H, d, *J* = 5.2 Hz, Ar*H*), 8.09-8.03 (2H, m, AA′BB′,2×Ar*H*), 7.81 (1H, d, *J* = 4.0 Hz, Ar*H*), 7.38 (1H, d, *J* = 5.2 Hz, Ar*H*), 7.29-7.23 (2H, m, AA′BB′, 2×Ar*H*), 6.66 (1H, d, *J* = 4.0 Hz, Ar*H*), 2.40 (3H, s, tosyl C*H₃*); *m/z* (ES^+) 351 ($C_{14}H_{11}{}^{79}BrN_2O_2S$, 100%, $[M]^+$), 353 ($C_{14}H_{11}{}^{81}BrN_2O_2S$, 100%, $[M]^+$).

The analytical data is consistent with the literature [3].

4-Bromo-2-iodo-1-tosyl-1*H*-pyrrolo[2,3-*b*]pyridine (3)

To a solution of 4-bromo-1-tosyl-1*H*-pyrrolo[2,3-*b*]pyridine, (**2**) (1.00 g, 2.86 mmol) and tetramethylethylenediamine (0.47 mL, 3.14 mmol) in THF (22.8 mL) was added a 0.76 M solution of LDA in THF (4.13 mL, 3.14 mmol) over a period of 5 min at -78°C. The resulting solution was allowed to warm to -50°C and then stirred between -50°C and -55°C for 60 min. The reaction mixture was then cooled to -78°C followed by the addition of solid iodine (1.09 g, 4.28 mmol) in one portion. The reaction mixture was allowed to warm to rt and stirred for an additional 16 h. The reaction was cooled to 0°C and quenched with satd. aq. NH_4Cl (20 mL) and extracted with EtOAc (3×25 mL). The combined organic layers were washed with aq. $Na_2S_2O_3$ (40 mL) and brine (40 mL), dried over $MgSO_4$, filtered and concentrated under reduced pressure to obtain the crude compound which was purified using flash column chromatography (80 g silica, 0:100 → 15:85 EtOAc:heptane) to obtain the title compound as a white solid (1.05 g, 2.19 mmol, 77%); ^1H NMR (500 MHz, $CDCl_3$) δ 8.18 (1H, d, *J* = 5.3 Hz, Ar*H*), 8.12-8.06 (2H, m, AA′BB′, 2×Ar*H*), 7.31 (1H, d, *J* = 5.3 Hz, Ar*H*), 7.30-7.27 (2H, m, AA′BB′, 2×Ar*H*), 7.04 (1H, s, Ar*H*), 2.38 (3H, s, tosyl C*H₃*); m/z (ES^+) 477 ($C_{14}H_{10}{}^{79}BrIN_2O_2S$, 100%, $[M]^+$), 479 ($C_{14}H_{10}{}^{81}BrIN_2O_2S$, 100%, $[M]^+$).

The analytical data is consistent with the literature [3].

1-(4-(4-Bromo-1-tosyl-1*H*-pyrrolo[2,3-*b*]pyridin-2-yl)phenyl)-*N*-methylmethanamine (4a)

To a solution of 4-bromo-2-iodo-1-tosyl-1*H*-pyrrolo[2,3-*b*]pyridine (**3**) (539 mg, 1.13 mmol), in 1,4-dioxane (3.8 mL) were added *N*-methyl-1-[4-(4,4,5,5-tetramethyl-1,3,2-dioxaborolan-2-yl)phenyl]methanamine (307 mg, 1.24 mmol), $Pd(PPh_3)_4$ (65 mg, 0.050 mmol) and a 2.0 M aq. soln. of K_3PO_4 (0.62 mL, 1.24 mmol). The flask was evacuated and purged with nitrogen thrice and then stirred at 110ºC for 16 h. The reaction mixture was subsequently concentrated under reduced pressure and the residue partitioned between EtOAc (20 mL) and water (15 mL). The aqueous layer was further extracted with additional EtOAc (2×20 mL). The combined organics were washed with brine (30 mL), dried over $MgSO_4$, filtered and concentrated under reduced pressure to obtain the crude product which was purified by flash column chromatography (24 g silica, 0:100 → 20:80 NH_3 in MeOH:CH_2Cl_2) to obtain the title compound (225 mg, 0.48 mmol, 42%) as a yellow solid; ^1H NMR (500 MHz, MeOD-*d₄*) δ 8.19 (1H, d, *J* = 5.3 Hz, Ar*H*), 7.65–7.59 (2H, m, AA′BB′, 2×Ar*H*), 7.56–7.52 (2H, m, AA′BB′, 2×Ar*H*), 7.50 (1H, d, *J* = 5.3 Hz, Ar*H*), 7.48–7.44 (2H, m, AA′BB′, 2×Ar*H*), 7.29–7.23 (2H, m, AA′BB′, 2×Ar*H*), 6.63 (1H, s, Ar*H*), 3.85 (2H, s, NC*H₂*), 2.47 (3H, s, NC*H₃*), 2.35 (3H, s, tosyl C*H₃*); ^{13}C NMR (126 MHz, MeOD-*d₄*) δ 149.3 (*C*), 145.8 (*C*), 144.4 (*C*H), 143.2 (*C*) 140.7 (*C*), 135.1 (*C*),

130.9 (*C*), 129.9 (*C*H), 129.2 (*C*H), 127.6 (*C*H), 126.9 (*C*), 127.3 (*C*H), 122.9 (*C*H), 109.9 (*C*), 108.1 (*C*H), 54.5 (*C*H_2), 34.1 (*C*H_3), 20.1 (*C*H_3); m/z (ES^+) 439 ($C_{21}H_{17}{}^{79}BrN_2O_2S$, 100%, $[M\text{-}NHMe]^+$), 441 ($C_{21}H_{17}{}^{81}BrN_2O_2S$, 100%, $[M\text{-}NHMe]^+$), 470 ($C_{22}H_{20}{}^{79}BrN_3O_2S$, 50%, $[M]^+$), 471 ($C_{22}H_{21}{}^{79}BrN_3O_2S$, 50%, $[M+H]^+$).

Note: some quaternary carbons were not visible in the ^{13}C-NMR spectrum and were assigned using the ^1H-^{13}C HMBC spectrum.

1-(4-(4-Bromo-1-tosyl-1*H*-pyrrolo[2,3-*b*]pyridin-2-yl)phenyl)-*N,N*-dimethylmethanamine (4b)

To a solution of 4-bromo-2-iodo-1-tosyl-1*H*-pyrrolo[2,3-*b*]pyridine (**3**) (479 mg, 1.00 mmol), in 1,4-dioxane (3 mL) were added *N,N*-dimethyl-1-[4-(4,4,5,5-tetramethyl-1,3,2-dioxaborolan-2-yl)phenyl]methanamine (288 mg, 1.10 mmol), $Pd(PPh_3)_4$ (58 mg, 0.050 mmol) and a 2.0 M aq. soln. of K_3PO_4 (0.55 mL, 1.10 mmol). The flask was then evacuated and purged with nitrogen thrice and then stirred at 110ºC for 16 h. The reaction mixture was subsequently concentrated under reduced pressure and the residue partitioned between EtOAc (15 mL) and water (15 mL). The aqueous layer was further extracted with additional EtOAc (2×15 mL). The combined organics were washed with brine (30 mL), dried over $MgSO_4$, filtered and concentrated under reduced pressure to obtain the crude compound which was purified by flash column chromatography (24 g silica, 0:100 → 20:80 NH_3 in MeOH:CH_2Cl_2) to obtain the title compound (287 mg, 0.59 mmol, 59%) as a yellow solid; ^1H NMR (500 MHz, MeOD-*d₄*) δ 8.19 (1H, d, *J* = 5.3 Hz, Ar*H*), 7.64–7.59 (2H, m, AA′BB′, 2×Ar*H*), 7.56-7.51 (2H, m, AA′BB′, 2×Ar*H*), 7.49 (1H, d, *J* = 5.3 Hz, Ar*H*), 7.46–7.41 (2H, m, AA′BB′, 2×Ar*H*), 7.28–7.23 (2H, m, AA′BB′, 2×Ar*H*), 6.63 (1H, s, Ar*H*), 3.59 (2H, s, NCH_2), 2.33 (3H, s, tosyl CH_3), 2.32 (6H, s, N*Me*$_2$); ^{13}C NMR (126 MHz, MeOD-d_4) δ 148.0 (*C*), 144.4 (*C*), 142.9 (*CH*), 141.7 (*C*), 137.2 (*C*), 133.7 (*C*), 129.7 (*C*), 128.3 (*C*H), 127.8 (*C*H), 127.2 (*C*H), 125.8 (*C*H), 123.3 (*C*), 122.5 (*C*), 121.4 (*C*H), 106.6 (*C*H), 61.8 (*C*H_2), 42.5 (*C*H_3), 22.2 (*C*H_3); m/z (ES^+) 439 ($C_{21}H_{17}{}^{79}BrN_2O_2S$, 100%, $[M\text{-}NHMe_2]^+$), 441 ($C_{21}H_{17}{}^{81}BrN_2O_2S$, 100%, $[M\text{-}NHMe_2]^+$), 485 ($C_{23}H_{23}{}^{79}BrN_3O_2S$, 20%, $[M+H]^+$), 487 ($C_{23}H_{23}{}^{81}BrN_3O_2S$, 20%, $[M+H]^+$).

1,1'-((1-Tosyl-1*H*-pyrrolo[2,3-*b*]pyridine-2,4-diyl)bis(4,1-phenylene))bis(*N,N*-dimethylmethanamine) (5)

To a solution of 1-(4-(4-bromo-1-tosyl-1*H*-pyrrolo[2,3-*b*]pyridin-2-yl)phenyl)-*N,N*-dimethylmethanamine (118 mg, 0.24 mmol) in degassed 1,4-dioxane (1.2 mL) was added *N,N*-dimethyl-1-[4-(4,4,5,5-tetramethyl-1,3,2-dioxaborolan-2-yl)phenyl]methanamine (66 mg, 0.27 mmol), Pd(dppf)Cl_2 (10 mg, 0.010 mmol), and Na_2CO_3 (52 mg, 0.49 mmol). The reaction mixture was heated in an inert atmosphere at 110°C for 16 h. The reaction mixture was cooled to rt partitioned between EtOAc (15 mL) and water (15 mL). The aqueous layer was further extracted with additional EtOAc (2x15 mL). The organics were combined and washed with brine (30 mL), dried over $MgSO_4$, filtered and loaded onto a column (30 g, C_{18}, wet-load in 1;1 MeCN:water); and purified by flash column chromatography (0:100 → 100:0 0.1% aq. HCOOH:MeCN) to obtain the title compound (58 mg, 0.11 mmol, 44%) as a yellow solid; ^1H NMR (500 MHz, MeOD-d_4) δ 8.44 (1H, d, *J* = 5.1 Hz, Ar*H*), 7.76–7.73 (2H, m, AA′BB′, 2×Ar*H*), 7.73–7.70 (2H, m, AA′BB′, 2×Ar*H*), 7.69–7.65 (2H, m, AA′BB′, 2×Ar*H*), 7.64–7.58 (4H, m, 2×AA′BB′, 4×Ar*H*), 7.41 (1 H, d, *J* = 5.1 Hz, Ar*H*), 7.29 – 7.25 (2H, m, AA′BB′, 2×Ar*H*), 6.86 (1H, s, Ar*H*), 4.30 (2H, s, NCH_2), 4.22 (2H, s, NCH_2), 2.83 (6H, s, N*Me*$_2$), 2.76 (6H, s, N*Me*$_2$), 2.34 (3H, s, tosyl CH_3)) ^{13}C NMR (126 MHz, MeOD-d_4) δ 150.7 (*C*), 145.5 (*C*), 144.3 (*C*H),

142.6 (*C*), 141.9 (*C*), 138.7 (*C*), 138.4 (*C*), 136.2 (*C*), 135.2 (*C*), 131.7 (*C*H), 130.0 (*C*H), 129.8 (*C*H), 129.2 (*C*H), 128.6 (*C*H), 128.2 (*C*), 127.2 (*C*H), 120.5 (*C*), 118.7 (*C*H), 108.5 (*C*H), 63.3 (CH_2), 63.1 (CH_2), 42.0 (CH_3), 41.9 (CH_3), 20.1 (CH_3); m/z (ES^+) 539 ($C_{32}H_{34}N_4O_2S$, 25%, $[M+H]^+$).

1,1'-((1*H*-pyrrolo[2,3-*b*]pyridine-2,4-diyl)bis(4,1-phenylene))bis(*N,N*-dimethylmethanamine) (6, MMV022224)

To a solution of 1,1'-((1-tosyl-1*H*-pyrrolo[2,3-*b*]pyridine-2,4-diyl)bis(4,1-phenylene))bis(*N,N*-dimethylmethanamine) (**5**) (58 mg, 0.11 mmol) in MeOH (0.6 mL) was added K_2CO_3 (52 mg, 0.38 mmol) and stirred at 55°C for 16 h. The reaction mixture was cooled to rt and diluted with MeOH (3 mL). An aliquot was taken and checked by LCMS to determine if all the starting material had been consumed. Solvent was removed under reduced pressure and then the crude reaction mixture was suspended in water (20 mL) and EtOAc (20 mL). The layers were separated, and the aqueous layer was acidified to pH = 7 using 1.0 M hydrochloric acid (aq) and extracted further with EtOAc (3×15 mL). The organics were combined, washed with brine (30 mL), dried over $MgSO_4$, filtered and concentrated under reduced pressure to obtain a yellow solid. The crude product was washed with ice-cold Et_2O (3×0.7 mL) to obtain the desired product as a yellow solid (20 mg, 0.052 mmol, 48%); ^1H NMR (500 MHz, MeOD-*d*$_4$) δ 8.23 (1H, d, *J* = 5.1 Hz, Ar*H*), 7.88-7.83 (2H, m, AA′BB′, 2×Ar*H*), 7.83–7.78 (2H, m, 2×AA′BB′, 2×Ar*H*), 7.56–7.50 (2H, m, 2×AA′BB′, 2×Ar*H*), 7.46–7.40 (2H, m, 2×AA′BB′, 2×Ar*H*), 7.21 (1H, d, *J* = 5.1, Ar*H*), 7.03 (1H, s, Ar*H*), 3.59 (2H, s, NCH_2), 3.53 (2H, s, NCH_2), 2.32 (6H, s, N*Me*$_2$), 2.28 (6H, s, N*Me*$_2$); ^{13}C NMR (126 MHz, MeOD-*d*$_4$) δ 142.4 (*C*H), 141.5 (*C*), 139.3 (*C*), 137.9 (*C*), 137.6 (*C*), 131.1 (*C*), 129.9 (*C*H), 129.8 (*C*H), 128.2 (*C*H), 125.3 (*C*H), 123.9 (*C*), 119.9 (*C*), 114.7 (*C*H), 96.4 (*C*H), 63.3 (CH_2), 63.2 (CH_2), 43.9 (CH_3), 43.8 (CH_3); m/z (ES^+) 385 ($C_{25}H_{29}N_4$, 10%, $[M+H]^+$); HRMS $[C_{25}H_{28}N_4+H]^+$ calcd. 385.2392, found 385.2387 (-1.3 ppm).

Note: some quaternary carbons were not visible in the ^{13}C-NMR spectrum due to low signal and were assigned using the ^{13}C-DEPT Q spectrum.

N,N-dimethyl-1-(4-(2-(4-((methylamino)methyl)phenyl)-1-tosyl-1*H*-pyrrolo[2,3-*b*]pyridin-4-yl)phenyl)methanamine (7a)

To a solution of 1-(4-(4-bromo-1-tosyl-1*H*-pyrrolo[2,3-*b*]pyridin-2-yl)phenyl)-*N*-methylmethanamine (225 mg, 0.48 mmol) in degassed 1,4-dioxane (2.4 mL) was added *N,N*-dimethyl-1-[4-(4,4,5,5-tetramethyl-1,3,2-dioxaborolan-2-yl)phenyl]methanamine (137 mg, 0.53 mmol), Pd(dppf)Cl_2 (20 mg, 0.020 mmol), and Na_2CO_3 (101 mg, 0.96 mmol). The reaction mixture was heated in an inert atmosphere at 110°C for 16 h. The reaction mixture was cooled to rt partitioned between EtOAc (15 mL) and water (15 mL). The aqueous layer was further extracted with additional EtOAc (2×15 mL). The organics were combined and washed with brine (30 mL), dried over $MgSO_4$, filtered and loaded onto a column (30 g, C_{18}, wet-load in 1:1 MeCN:water); and purified by flash column chromatography (0:100 → 100:0 MeCN:0.1% aq. HCOOH) to obtain the title compound (167 mg, 0.32 mmol, 67%) as a yellow solid; ^1H NMR (500 MHz, MeOD-*d*$_4$) δ 8.44 (1H, d, *J* = 5.1 Hz, Ar*H*), 7.74–7.69 (4H, m, 2×AA′BB′, 4×Ar*H*), 7.68–7.64 (2H, m, AA′BB′, 2×Ar*H*), 7.62–7.58 (4H, m, 2×AA′BB′, 4×Ar*H*), 7.41 (1H, d, *J* = 5.1 Hz, Ar*H*), 7.30–7.24 (2H, m, AA′BB′, 2×Ar*H*), 6.84 (1H, s, Ar*H*), 4.28 (2H, s, NCH_2), 4.10 (2H, s, NCH_2), 2.78 (3H, s, NCH_3), 2.68 (6H, s, N*Me*$_2$), 2.34 (3H, s, tosyl CH_3); ^{13}C NMR (126 MHz, MeOD-*d*$_4$) δ 167.3 (*C*), 150.6 (*C*), 145.7 (*C*), 144.8 (*C*H), 142.1 (*C*), 141.9 (*C*), 138.0 (*C*), 135.1 (*C*), 134.0 (*C*), 132.0 (*C*H), 131.0 (*C*H), 130.5 (*C*H), 129.2 (*C*H), 128.8 (2x*C*H), 127.3 (*C*H),

120.3 (*C*), 118.9 (*C*H), 108.8 (*C*H), 61.2 (CH_2), 52.0 (CH_2), 42.3 (CH_3), 31.9 (CH_3), 20.1 (CH_3); m/z (ES$^+$) 526 ($C_{31}H_{34}N_4O_2S$, 50%, $[M+H]^+$).

***N,N*-dimethyl-1-(4-(4-(4-((methylamino)methyl)phenyl)-1-tosyl-1*H*-pyrrolo[2,3-*b*]pyridin-2-yl)phenyl)methanamine (7b)**

To a solution of 1-(4-(4-bromo-1-tosyl-1*H*-pyrrolo[2,3-*b*]pyridin-2-yl)phenyl)-*N,N*-dimethylmethanamine (208 mg, 0.43 mmol) in degassed 1,4-dioxane (2.1 mL) was added *N*-methyl-1-(4-(4,4,5,5-tetramethyl-1,3,2-dioxaborolan-2-yl)phenyl)methanamine (117 mg, 0.47 mmol), Pd(dppf)Cl_2 (18 mg, 0.020 mmol), and Na_2CO_3 (91 mg, 0.86 mmol). The reaction mixture was heated in an inert atmosphere at 110°C for 16 h. The reaction mixture was cooled to rt partitioned between EtOAc (15 mL) and water (15 mL). The aqueous layer was further extracted with additional EtOAc (2×15 mL). The organics were combined and washed with brine (30 mL), dried over $MgSO_4$, filtered and loaded onto a column (30 g, C_{18}, wet-load in 1:1 MeCN:water); and purified by flash column chromatography (0:100 → 100:0 MeCN/0.1% aq. HCOOH) to obtain the title compound (148 mg, 0.28 mmol, 66%) as a yellow solid; ^{1}H NMR (500 MHz, MeOD-*d₄*) δ 8.44 (1H, d, *J* = 5.1 Hz, Ar*H*), 7.77–7.73 (2H, m, AA′BB′, 2×Ar*H*), 7.70–7.65 (4H, m, 2×AA′BB′, 4×Ar*H*), 7.63–7.59 (2H, m, AA′BB′, 2×Ar*H*), 7.58–7.53 (2H, m, 2×Ar*H*), 7.41 (1H, d, *J* = 5.1, Ar*H*), 7.30–7.25 (2H, m, 2×AA′BB′, 2×Ar*H*), 6.81 (1H, s, Ar*H*), 4.23 (2H, s, NCH_2), 4.12 (2H, s, NCH_2), 2.73 (3H, s, NCH_3), 2.71 (6H, s, N*Me₂*), 2.34 (3H, s, tosyl CH_3); ^{13}C NMR (126 MHz, MeOD-d_4) δ 151.6 (*C*), 144.7 (*C*H), 145.8 (*C*), 142.2 (*C*), 135.5 (*C*), 133.1 (*C*), 130.3 (*C*H), 130.2 (*C*H), 129.6 (*C*), 129.5 (*C*H), 129.2 (*C*H), 129.0 (*C*H), 127.3 (*C*H), 121.0 (*C*), 118.8 (*C*H), 108.4 (*C*H), 61.5 (CH_2), 51.9 (CH_2), 42.5 (CH_3), 31.9 (CH_3), 20.1 (CH_3); *m/z* (ES$^+$) 526 ($C_{31}H_{34}N_4O_2S$, 50%, $[M+H]^+$).

Note: some quaternary carbons were not visible in the ^{13}C-NMR spectrum and were assigned using the ^{1}H-^{13}C HMBC spectrum.

***Tert*-butyl (2-(2-(2-((4-(4-(4-((dimethylamino)methyl)phenyl)-1*H*-pyrrolo[2,3-*b*]pyridin-2-yl)benzyl)(methyl)amino)ethoxy)ethoxy)ethyl)carbamate (8a)**

To a solution of *N,N*-dimethyl-1-(4-(2-(4-((methylamino)methyl)phenyl)-1-tosyl-1*H*-pyrrolo[2,3-*b*]pyridin-4-yl)phenyl)methanamine (**7a**) (170 mg, 0.32 mmol) and 2,2-dimethyl-4-oxo-3,8,11-trioxa-5-azatridecan-13-yl 4-methylbenzenesulfonate (144 mg, 0.36 mmol) in EtOH (1.4 mL) was added Na_2CO_3 (38 mg, 0.34 mmol), and the mixture heated to reflux for 16 h. Analysis by LCMS indicated that all the azaindole starting material had been used up. The reaction mixture was concentrated under reduced pressure and water (10 mL) added followed by extraction with EtOAc (3×15 mL). The organics were combined, washed with brine (20 mL), dried over $MgSO_4$, filtered and concentrated under reduced pressure. The material was suspended in MeOH (1.8 mL) and K_2CO_3 (90 mg, 0.64 mmol) was added, and the mixture heated at 65°C for 16 h. The reaction mixture was concentrated under reduced pressure to dryness and then loaded onto a C_{18} column (30 g, wet-load in MeOH); and purified by flash column chromatography (5:95 → 100:0 MeOH:0.1% aq. NH_3) to obtain the title compound (30 mg, 0.05 mmol, 15% over 2 steps) as a yellow oil; ^{1}H NMR (500 MHz, MeOD-d_4) δ 8.23 (1H, d, *J* = 5.1 Hz, Ar*H*), 7.87–7.83 (2H, m, AA′BB′, 2×Ar*H*), 7.83–7.78 (2H, m, AA′BB′, 2×Ar*H*), 7.55–7.50 (2H, m, AA′BB′, 2×Ar*H*), 7.48–7.42 (2H, m, AA′BB′, 2×Ar*H*), 7.20 (1H, d, *J* = 5.1 Hz, Ar*H*), 7.01 (1H, s, Ar*H*), 3.68 – 3.65 (2H, m, CH_2), 3.63 (2H, s, NCH_2), 3.61–3.59 (2H, m, CH_2), 3.57 (2H, s, CH_2), 3.52–3.49 (2H, m, NCH_2), 3.22–3.18 (4H, m, 2×CH_2), 2.69–2.64 (2H, m, CH_2), 2.31 (6H, s, NMe_2), 2.30 (3H, s, N*Me*), 1.40 (9H, s, ^{t}Bu-Me_3); ^{13}C NMR (126 MHz, MeOD-d_4)

δ 149.8 (C), 142.3 (*C*H), 141.5 (C), 139.4 (C), 138.3 (C), 138.0 (C), 137.9 (C), 133.6 (C), 130.8 (C), 129.9 (*C*H), 129.8 (*C*H), 128.2 (*C*H), 125.3 (*C*H), 119.9 (C), 114.7 (*C*H), 96.3 (*C*H), 78.8 (*C*), 70.0 (CH_2), 69.9 (CH_2), 69.7 (CH_2), 68.8 (CH_2), 63.3 (CH_2), 61.7 (CH_2), 55.9 (CH_2), 44.0 (CH_3), 41.7 (CH_3), 39.9 (CH_2), 27.4 (CH_3); m/z (ES^+) 602 ($C_{35}H_{47}N_5O_4$+H 100%, $[M+H]^+$); HRMS $[C_{35}H_{47}N_5O_4+H]^+$ calcd. 602.3706, found 602.3704 (-0.3 ppm).

Note: the ^{t}-butyl quaternary carbon was not visible in the ^{13}C-NMR and was assigned using the ^{1}H-^{13}C HMBC spectrum.

Tert-butyl (2-(2-(2-((4-(2-(4-((dimethylamino)methyl)phenyl)-1*H*-pyrrolo[2,3-*b*]pyridin-4-yl)benzyl)(methyl)amino)ethoxy)ethoxy)ethyl)carbamate (8b)

To a solution of *N,N*-dimethyl-1-(4-(4-(4-((methylamino)methyl)phenyl)-1-tosyl-1*H*-pyrrolo[2,3-*b*]pyridin-4-yl)phenyl)methanamine (**7b**) (128 mg, 0.24 mmol) and 2,2-dimethyl-4-oxo-3,8,11-trioxa-5-azatridecan-13-yl 4-methylbenzenesulfonate (108 mg, 0.27 mmol) in EtOH (1.1 mL) was added sodium carbonate (28 mg, 0.27 mmol), and the mixture heated to reflux for 16 h. Analysis by LCMS indicated that all the azaindole starting material had been used up. The reaction mixture was concentrated under reduced pressure and water (10 mL) added followed by extraction with EtOAc (3x15 mL). The organics were combined, washed with brine (20 mL), dried over $MgSO_4$, filtered and concentrated under reduced pressure. The material was suspended in MeOH (1.8 mL) and K_2CO_3 (67 mg, 0.49 mmol) was added, and the mixture heated at 65°C for 16 h. The reaction mixture was concentrated under reduced pressure to dryness and then loaded onto a column (30 g, C_{18}, wet-load in MeCN); and purified by flash column chromatography (5:95 → 100:0 MeCN:0.1% aq. NH_3) to obtain the title compound (13 mg, 0.02 mmol, 8% over 2 steps) as a yellow oil; ^{1}H NMR (500 MHz, MeOD-*d₄*) δ 8.23 (1H, d, *J* = 5.1 Hz, Ar*H*), 7.88–7.83 (2H, m, AA′BB′, 2×Ar*H*), 7.81–7.78 (2H, m, AA′BB′, 2×Ar*H*), 7.57–7.52 (2H, m, AA′BB′, 2×Ar*H*), 7.46–7.38 (2H, m, AA′BB′, 2×Ar*H*), 7.21 (1H, d, *J* = 5.1 Hz, Ar*H*), 7.03 (1H, s, Ar*H*), 3.70–3.68 (4H, m, 2×CH_2), 3.62 (4H, s, 2×NCH_2), 3.52–3.50 (4H, m, 2×CH_2), 3.23–3.20 (2H, m, CH_2), 2.72–2.67 (2H, m, CH_2), 2.35 (3H, s, N*Me*), 2.27 (6H, s, NMe_2), 1.40 (9H, s, ^{t}Bu-Me_3); ^{13}C NMR (126 MHz, MeOD-d_4) δ 149.8 (*C*), 142.3 (*C*H), 141.6 (*C*), 140.6 (*C*), 139.3 (*C*), 138.6 (*C*), 137.7 (*C*), 131.0 (*C*), 129.9 (*C*H), 129.8 (*C*H), 128.1 (*C*H), 125.3 (*C*H), 119.9 (*C*), 114.7 (*C*H), 96.5 (*C*H), 78.8 (*C*), 70.1 (CH_2), 69.9 (CH_2), 69.7 (CH_2), 68.8 (CH_2), 63.2 (CH_2), 61.8 (CH_2), 55.9 (CH_2), 43.9 (CH_3), 41.8 (CH_3), 27.4 (CH_3); m/z (ES^+) 602 ($C_{35}H_{47}N_5O_4$+H 100%, $[M+H]^+$); HRMS $[C_{35}H_{47}N_5O_4+H]^+$ calcd. 602.3706, found 602.3698 (-1.3 ppm).

Note: the ^{t}-butyl quaternary carbon was not visible in the ^{13}C-NMR and was assigned using the ^{1}H-^{13}C HMBC spectrum.

2-(2-(2-Aminoethoxy)ethoxy)-*N*-(4-(4-(4-((dimethylamino)methyl)phenyl)-1*H*-pyrrolo[2,3-*b*]pyridin-2-yl)benzyl)-*N*-methylethan-1-amine (9a)

To *tert*-butyl (2-(2-(2-((4-(4-(4-((dimethylamino)methyl)phenyl)-1*H*-pyrrolo[2,3-*b*]pyridin-2-yl)benzyl)(methyl)amino)ethoxy)ethoxy)ethyl)carbamate (**8a**) (16 mg, 0.027 mmol) was added an ice-cold solution of 1:2 TFA:CH_2Cl_2 (3 mL). The mixture was allowed to warm up to rt and stir for 2 h. The reaction mixture was concentrated under reduced pressure and then loaded onto an SCX cartridge in a minimum volume of MeOH. The SCX cartridge was washed with 3 column volumes of MeOH, followed by 3 column volumes of 7 N ammonia in MeOH, to elute the starting material as a free amine (12 mg, 0.023 mmol, 86%) as a yellow oil; ^{1}H NMR (500 MHz, MeOD-d_4) δ 8.23 (1H, d, *J* = 5.1, Ar*H*), 7.86–7.82 (2H, m, AA′BB′, 2×Ar*H*), 7.81–7.76

(2H, m, AA′BB′, 2×Ar*H*), 7.54–7.49 (2H, m, AA′BB′, 2×Ar*H*), 7.46–7.41 (2H, m, AA′BB′, 2×Ar*H*), 7.21 (1H, d, *J* = 5.1 Hz, Ar*H*), 7.02 (1H, s, Ar*H*), 3.68–3.64 (2H, m, C*H*$_2$), 3.64–3.61 (6H, m, 3×C*H*$_2$), 3.58 (2H, s, NC*H*$_2$), 3.56–3.52 (2H, m, C*H*$_2$), 2.86–2.80 (2H, m, C*H*$_2$), 2.68–2.62 (2H, m, C*H*$_2$), 2.31 (6H, s, N*Me*$_2$), 2.30 (3H, s, N*Me*); ^{13}C NMR (126 MHz, MeOD-*d*$_4$) δ 151.2 (*C*), 143.7 (*C*), 142.9 (*C*H), 140.8 (*C*), 139.6 (*C*), 139.4 (*C*), 139.3 (*C*), 132.2 (*C*), 131.3 (*C*H), 131.2 (*C*H), 129.6 (*C*H), 126.6 (*C*H), 121.3 (*C*), 116.1 (*C*H), 97.7 (*C*H), 72.5 (*C*H$_2$), 71.4 (*C*H$_2$), 71.3 (*C*H$_2$), 70.2 (*C*H$_2$), 64.7 (*C*H$_2$), 63.1 (*C*H$_2$), 57.3 (*C*H$_2$), 45.3 (*C*H$_3$), 43.1 (*C*H$_3$), 41.9 (*C*H$_2$); m/z (ES$^+$) 502 ($C_{30}H_{40}N_5O_2$, 100%, [M+H]$^+$); HRMS [$C_{30}H_{39}N_5O_2$+H]$^+$ calcd. 502.3182, found 502.3179 (-0.6 ppm).

2-(2-(2-Aminoethoxy)ethoxy)-*N*-(4-(2-(4-((dimethylamino)methyl)phenyl)-1*H*-pyrrolo[2,3-*b*]pyridin-4-yl)benzyl)-*N*-methylethan-1-amine (9b)

To an ice-cold solution of *tert*-butyl (2-(2-(2-((4-(2-(4-((dimethylamino)methyl)phenyl)-1*H*-pyrrolo[2,3-*b*]pyridin-4-yl)benzyl)(methyl)amino)ethoxy)ethoxy)ethyl)carbamate (**8b**) (10 mg, 16.6 nmol) in CH_2Cl_2 (1 mL) was added 4.0 M HCl in 1,4-dioxane (17 µL, 66.4 nmol) and the reaction mixture left to stir at rt for 16 h. The reaction mixture was concentrated under reduced pressure and purified by SCX cartridge to isolate the free amine product, **9b** (5 mg, 9.8 nmol, 59%) as a yellow oil; ^{1}H NMR (500 MHz, MeOD-*d*$_4$) δ 8.23 (1H, d, *J* = 5.1 Hz, Ar*H*), 7.87–7.81 (2H, m, AA′BB′, 2×Ar*H*), 7.81–7.74 (2H, m, AA′BB′, 2×Ar*H*), 7.56–7.50 (2H, m, AA′BB′, 2×Ar*H*), 7.44–7.39 (2H, m, AA′BB′, 2×Ar*H*), 7.20 (1H, d, *J* = 5.1 Hz, Ar*H*), 7.02 (1H, s, Ar*H*), 3.70–3.66 (4H, m, C*H*$_2$, NC*H*$_2$), 3.65–3.62 (4H, m, 2×C*H*$_2$), 3.56–3.53 (2H, m, C*H*$_2$), 3.51 (2H, s, NC*H*$_2$), 2.85–2.79 (2H, m, C*H*$_2$), 2.71–2.65 (2H, m, C*H*$_2$), 2.33 (3H, s, N*Me*), 2.27 (6H, s, N*Me*$_2$); ^{13}C NMR (126 MHz, MeOD-*d*$_4$) δ 151.3 (*C*), 143.8 (*CH*), 143.0 (*C*), 140.7 (*C*), 139.9 (*C*), 139.1 (*C*), 139.0 (*C*), 132.4 (*C*), 131.3 (*C*H), 131.2 (*C*H), 129.5 (*C*H), 126.7 (*C*H), 121.2 (*C*), 116.1 (*C*H), 97.9 (*C*H), 72.4 (*C*H$_2$), 71.4 (2x*C*H$_2$), 70.2 (*C*H$_2$), 64.6 (*C*H$_2$), 63.1 (*C*H$_2$), 57.3 (*C*H$_2$), 45.3 (*C*H$_3$), 43.2 (*C*H$_3$) 41.9 (*C*H$_2$); m/z (ES$^+$) 502 ($C_{30}H_{40}N_5O_2$, 100%, [M+H]$^+$); HRMS [$C_{30}H_{39}N_5O_2$+H]$^+$ calcd. 502.3182, found 502.3164 (-3.6 ppm).

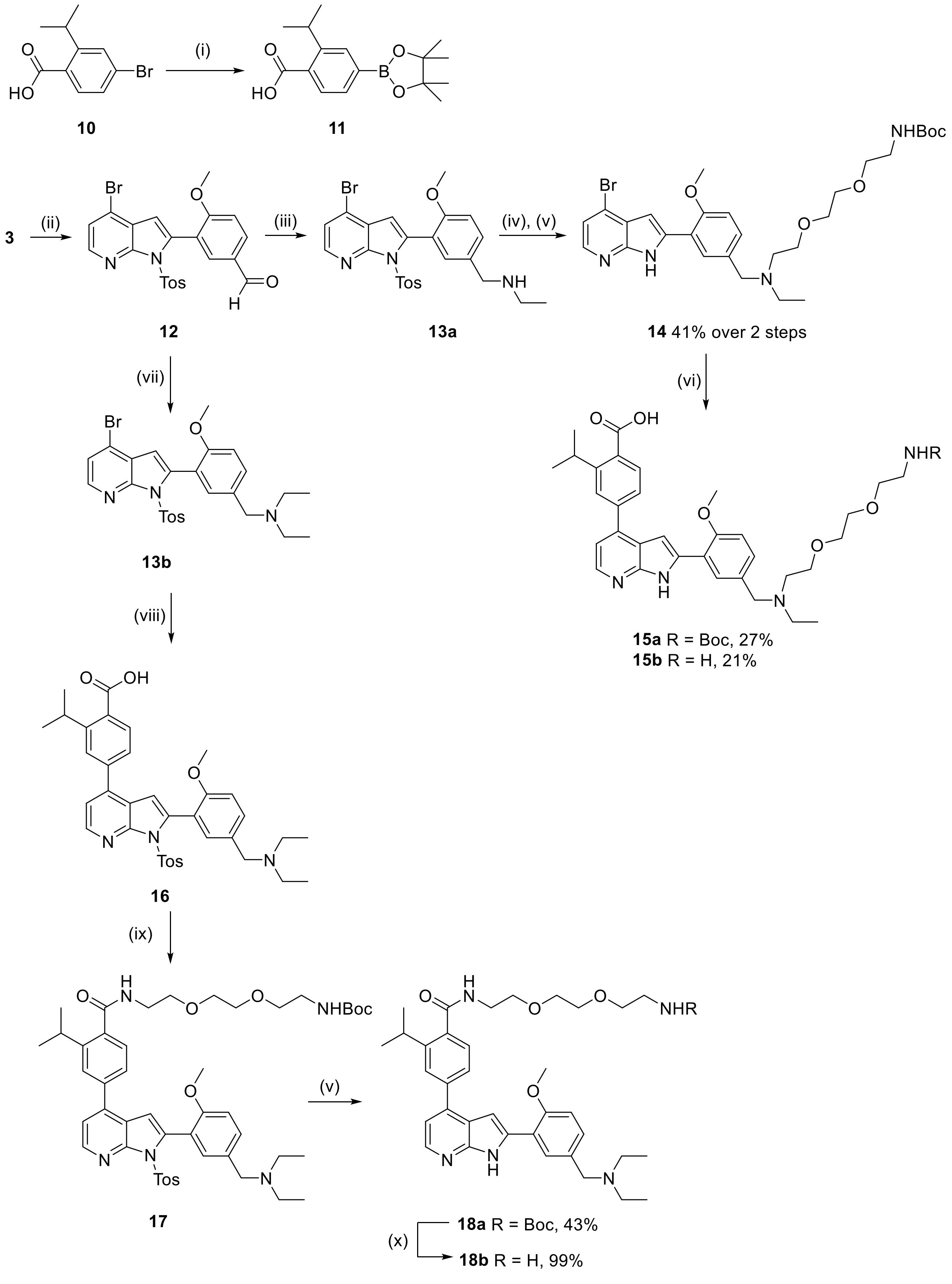

Scheme S3. Synthesis of TCMDC-135051-based probes.

Reagents and conditions – (i) B(pin)$_2$, Pd(dppf)Cl$_2$, K_2CO_3, 1,4-dioxane, 100°C, 16 h, 96%; (ii) (5-formyl-2-methoxy-phenyl)boronic acid, Pd(PPh$_3$)$_4$, Na_2CO_3, 1,4-dioxane, 100°C, 18 h, 79%; (iii) EtNH$_2$, AcOH, NaBH(OAc)$_3$, 1,4-dioxane, rt, 18 h, 78%; (iv) 2,2-dimethyl-4-oxo-3,8,11-trioxa-5-azatridecan-13-yl methanesulfonate, Na_2CO_3, EtOH, 78°C, 16 h; (v) K_2CO_3, MeOH, 65°C, 16 h; (vi) **11**, Pd(dppf)Cl$_2$, Na_2CO_3 (aq), 1,4-dioxane, 100°C, 16 h, 27% **15a**, 21% **15b**; (vii) Et$_2$NH, AcOH, NaBH(OAc)$_3$, 1,4-dioxane, rt, 18 h, 78%; (viii) **11**, Pd(dppf)Cl$_2$, Na_2CO_3 (aq), 1,4-dioxane, 100°C, 16 h, 42%; (ix) *tert*-butyl (2-(2-(2-aminoethoxy)ethoxy)ethyl)carbamate, DIPEA, HCTU, DMF, rt, 16 h, 97%; (x) TFA, CH_2Cl_2, rt, 2 h, 99%.

2,2-dimethyl-4-oxo-3,8,11-trioxa-5-azatridecan-13-yl methanesulfonate

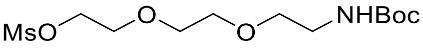

Prepared from *tert*-butyl (2-(2-(2-hydroxyethoxy)ethoxy)ethyl)carbamate using a literature method (*Eur. J. Org. Chem*. 2011, 1641–1644).

2-Isopropyl-4-(4,4,5,5-tetramethyl-1,3,2-dioxaborolan-2-yl)benzoic acid (11)

Prepared from 4-bromo-2-isopropyl-benzoic acid (**10**) using a modification of a literature method [3].

3-(4-Bromo-1-tosyl-1*H*-pyrrolo[2,3-*b*]pyridin-2-yl)-4-methoxybenzaldehyde (12)

Prepared from 4-bromo-2-iodo-1-(p-tolylsulfonyl)pyrrolo[2,3-b]pyridine (**3**) according to the literature method [3].

***N*-(3-(4-Bromo-1-tosyl-1*H*-pyrrolo[2,3-*b*]pyridin-2-yl)-4-methoxybenzyl)ethanamine (13a)**

A solution of 3-(4-bromo-1-tosyl-1*H*-pyrrolo[2,3-*b*]pyridin-2-yl)-4-methoxybenzaldehyde (**12**) (1.27 g, 2.61 mmol), AcOH (0.30 mL, 5.23 mmol), and 30% ethylamine solution (2.61 mL, 5.23 mmol) in 1,4-dioxane (23 mL) was stirred for 1 h at rt, before the addition of NaBH(OAc)$_3$ (1.38 g, 6.53 mmol). The reaction mixture was stirred at rt for a further 18 h, then diluted with aqueous $NaHCO_3$ (20 mL) and extracted with EtOAc (3×30 mL). The combined organic layers were washed with brine (30 mL), dried over Na_2SO_4, filtered and concentrated under reduced pressure to give a residue that was purified by flash column chromatography (24 g silica, 0:10:90 → 10:90:0 MeOH:EtOAc:heptane) to obtain *N*-(3-(4-bromo-1-tosyl-1*H*-pyrrolo[2,3-*b*]pyridin-2-yl)-4-methoxybenzyl)ethanamine (**13a**) (1.04 g, 2.02 mmol, 78%) as a white solid; ^{1}H NMR (500 MHz, CDCl$_3$) δ 8.20 (1H, dd, *J* = 5.2, 1.2 Hz, Ar*H*), 7.91–7.82 (2H, m, AA′BB′, 2×Ar*H*), 7.45 (1H, dd, *J* = 8.4, 2.2 Hz, Ar*H*), 7.39–7.29 (2H, m, 2×Ar*H*), 7.23–7.15 (2H, m, AA′BB′, 2×Ar*H*), 6.94 (1H, d, *J* = 8.4 Hz, Ar*H*), 6.54 (1H, s, Ar*H*), 3.84 (2H, s, C*H$_2$*), 3.79 (3H, s, OC*H$_3$*), 2.76 (2H, q, *J* = 7.2 Hz, NC*H$_2$*), 2.35 (3H, s, tosyl C*H$_3$*), 1.19 (3H, t, *J* = 7.2 Hz, ethyl C*H$_3$*); ^{13}C NMR (126 MHz, CDCl$_3$) δ 157.4 (*C*), 148.8 (*C*), 144.7 (*C*), 144.3 (*C*H), 139.3 (*C*), 136.2 (*C*), 131.0 (*C*H), 130.8 (*C*H), 129.2 (*C*H), 129.1 (*C*), 128.2 (*C*H), 124.7 (*C*), 123.5 (*C*), 122.1 (*C*H), 121.7 (*C*), 110.4 (*C*H), 107.4 (*C*H), 55.6 (*C*H$_3$), 53.0 (*C*H$_2$), 43.5 (*C*H$_2$), 21.6 (*C*H$_3$), 15.0 (*C*H$_3$); m/z (ES$^+$) 515 ($C_{24}H_{24}{}^{79}BrN_3O_3S$, 50%, [M]$^+$), 517 ($C_{14}H_{10}{}^{81}BrIN_2O_2S$, 50%, [M]$^+$).

_N_-(3-(4-Bromo-1-tosyl-1_H_-pyrrolo[2,3-_b_]pyridin-2-yl)-4-methoxybenzyl)-_N_-ethylethanamine (13b)

Prepared from 3-(4-bromo-1-tosyl-1H-pyrrolo[2,3-b]pyridin-2-yl)-4-methoxybenzaldehyde (**12**) according to the literature method [3].

Tert-butyl (2-(2-(2-((3-(4-bromo-1_H_-pyrrolo[2,3-_b_]pyridin-2-yl)-4-methoxybenzyl)(ethyl)amino)ethoxy)ethoxy)ethyl)carbamate (14)

To a solution of *N*-(3-(4-bromo-1-tosyl-1*H*-pyrrolo[2,3-*b*]pyridin-2-yl)-4-methoxybenzyl)ethanamine (**13a**) (1.05 g, 2.04 mmol) and 2,2-dimethyl-4-oxo-3,8,11-trioxa-5-azatridecan-13-yl methanesulfonate (734 mg, 2.24 mmol) in EtOH (11.3 mL) was added Na_2CO_3 (432 mg, 4.08 mmol), and the mixture heated to reflux for 16 h. The mixture was concentrated under reduced pressure, diluted in MeOH and analysed by LCMS. LCMS showed consumption of secondary amine **13a** and the presence of product (*m/z* 745, $[M]^+$) in the crude mixture. The reaction mixture was further worked up by concentrating under reduced pressure and adding water (20 mL), followed by extraction with EtOAc (3×20 mL). The organics were combined, washed with brine (20 mL) dried over $MgSO_4$, filtered and concentrated under reduced pressure to give the crude product which was used in the next step without further purification.

The crude tertiary amine was dissolved in MeOH (11.2 mL) and solid K_2CO_3 (564 mg, 4.08 mmol) added the resultant suspension was stirred at 65ºC for 16 h. The reaction mixture was concentrated under reduced pressure and the residue purified by reversed-phase flash column chromatography (150 g C_{18}, 5:95 → 100:0 MeCN:0.1% aq. NH_3) to obtain the title compound (494 g, 0.83 mmol, 41% over 2 steps) as a yellow solid; ^{1}H NMR (500 MHz, MeOD-*d₄*) δ 8.08 (1H, d, *J* = 5.2 Hz, Ar*H*), 7.90 (1H, d, *J* = 2.2 Hz, Ar*H*), 7.41 (1H, dd, *J* = 8.4, 2.2 Hz, Ar*H*), 7.36 (1H, d, *J* = 5.2 Hz, Ar*H*), 7.17 (1H, d, *J* = 8.4 Hz, Ar*H*), 6.99 (1H, s, Ar*H*), 4.07 (3H, s, OCH_3), 3.73 (2H, s, ArCH_2N), 3.70–3.66 (2H, m, CH_2), 3.66–3.61 (4H, m, 2xCH_2), 3.57–3.50 (2H, m, CH_2), 3.28–3.23 (2H, m, CH_2), 2.80–2.75 (2H, m, CH_2), 2.71 (2H, q, *J* = 7.1 Hz, ethyl CH_2), 1.48 (9H, s, ^{t}Bu-*Me₃*), 1.18 (3H, t, *J* = 7.1 Hz, ethyl CH_3); ^{13}C NMR (126 MHz, MeOD-*d₄*) δ 154.4 (*C*), 146.5 (*C*), 140.6 (*C*H), 135.8 (*C*), 130.1 (*C*), 129.2 (*C*H), 127.7 (*C*H), 122.6 (*C*), 121.1 (*C*), 117.6 (*C*), 117.3 (*C*H), 110.0 (*C*H), 97.2 (*C*H), 77.2 (*C*), 68.5 (CH_2), 68.4 (CH_2), 68.2 (CH_2), 67.5 (CH_2), 55.9 (CH_2), 53.5 (CH_3), 50.4 (CH_2), 46.2 (CH_2), 38.4 (CH_2), 25.9 (CH_3), 9.0 (CH_3); m/z (ES^+) 593 ($C_{28}H_{39}{}^{79}BrN_4O_5$, 70%, $[M]^+$), 595 ($C_{28}H_{39}{}^{81}BrN_4O_5$, 70%, $[M]^+$).

Note: one of the aromatic quaternary carbons was not visible in the ^{13}C-NMR

4-(2-(5-(2-Ethyl-14,14-dimethyl-12-oxo-5,8,13-trioxa-2,11-diazapentadecyl)-2-methoxyphenyl)-1_H_-pyrrolo[2,3-_b_]pyridin-4-yl)-2-isopropylbenzoic acid (15a) and 4-(2-(5-(((2-(2-(2-aminoethoxy)ethoxy)ethyl)(ethyl)amino)methyl)-2-methoxyphenyl)-1_H_-pyrrolo[2,3-_b_]pyridin-4-yl)-2-isopropylbenzoic acid (15b)

To a mixture of *tert*-butyl (2-(2-(2-((3-(4-bromo-1*H*-pyrrolo[2,3-*b*]pyridin-2-yl)-4-methoxybenzyl)(ethyl)amino)ethoxy)ethoxy)ethyl)carbamate (**14**) (492 mg, 0.83 mmol), 2-isopropyl-4-(4,4,5,5-tetramethyl-1,3,2-dioxaborolan-2-yl)benzoic acid (**11**) (265 mg, 0.91 mmol), in 1,4-dioxane (6.4 mL) was added Pd(dppf)Cl_2 (67.9 mg, 0.083 mmol) and Na_2CO_3 (264 mg, 2.5 mmol) in water (1.4 mL). The mixture was degassed with N_2 thrice and then

stirred at 100°C for 16 h. The reaction mixture was diluted with water (20 mL) and the mixture was adjusted to approximately pH 3 with HCl (1.0 M, aq), followed by extraction with EtOAc (3×30 mL). The combined organic layers were washed with brine (30 mL), dried over $MgSO_4$, filtered and concentrated under reduced pressure. LCMS analysis of the aqueous layer revealed the presence of product, therefore, the aqueous was also concentrated under reduced pressure. The combined crude from both layers was purified by reversed phase prep HPLC (Column: Waters Xbridge 100×19 mm, 5 µm particle size; mobile phase: 5:95 → 100:0 MeCN:0.1% NH_3 (aq)) to give both the free amine 4-(2-(5-(((2-(2-(2-aminoethoxy)ethoxy)ethyl)(ethyl)amino)methyl)-2-methoxyphenyl)-1*H*-pyrrolo[2,3-*b*]pyridin-4-yl)-2-isopropylbenzoic acid (**15b**) (100 mg, 0.17 mmol, 21%), which eluted before the protected amine 4-(2-(5-(2-ethyl-14,14-dimethyl-12-oxo-5,8,13-trioxa-2,11-diazapentadecyl)-2-methoxyphenyl)-1*H*-pyrrolo[2,3-*b*]pyridin-4-yl)-2-isopropylbenzoic acid (**15a**) (150mg, 0.22 mmol, 27%).

Analytical data for 4-(2-(5-(2-ethyl-14,14-dimethyl-12-oxo-5,8,13-trioxa-2,11-diazapentadecyl)-2-methoxyphenyl)-1*H*-pyrrolo[2,3-*b*]pyridin-4-yl)-2-isopropylbenzoic acid (**15a**):

^{1}H NMR (500 MHz, MeOD-*d₄*) δ 8.26 (1H, d, *J* = 5.1 Hz, Ar*H*), 7.97 (1H, s, Ar*H*), 7.73 (1H, s, Ar*H*), 7.59–7.55 (2H, m, 2×Ar*H*), 7.48 (1H, d, *J* = 8.5 Hz, Ar*H*), 7.25 (1H, d, *J* = 8.5 Hz, Ar*H*), 7.22 (1H, d, *J* = 5.1 Hz, Ar*H*), 7.14 (1H, s, Ar*H*), 4.26 (2H, s, ArCH_2N), 4.05 (3H, s, OCH_3), 3.83–3.76 (2H, m, OCH_2), 3.74–3.67 (1H, m, isopropyl C*H*), 3.63 (4H, m, 2×CH_2), 3.47–3.42 (2H, m, OCH_2), 3.26–3.21 (2H, m, OCH_2), 3.20–3.11 (4H, m, 2×CH_2), 1.51–1.25 (18H, isopropyl 2×CH_3, ethyl CH_3, ^{t}Bu-*Me*$_3$); ^{13}C NMR (126 MHz, MeOD-d_4) δ 176.1 (*C*), 157.3 (*C*), 148.9 (*C*), 146.1 (*C*), 142.6 (*C*H), 142.2 (*C*), 139.5 (*C*), 138.5 (*C*), 135.7 (*C*), 131.7 (*C*H), 130.5 (*C*H), 127.3 (*C*H), 125.3 (*C*H), 125.1 (*C*H), 124.2 (*C*), 120.6 (*C*), 119.1 (*C*), 114.6 (*C*H), 112.1 (*C*H), 99.3 (*C*H), 78.7 (*C*), 70.0 (CH_2), 69.8 (CH_2), 69.6 (CH_2), 65.5 (CH_2), 56.6 (CH_2), 55.1 (CH_3), 51.1 (CH_2), 47.9 (CH_2), 39.8 (CH_2), 29.9 (*C*H), 27.3 (CH_3), 23.2 (CH_3), 8.4 (CH_3); m/z (ES^+) 675 ($C_{38}H_{51}N_4O_7$, 100%, $[M+H]^+$); HRMS $[C_{38}H_{50}N_4O_7+H]^+$ calcd. 675.3758, found 675.3728 (-4.4 ppm).

Note: one of the aromatic quaternary carbons was not visible in the ^{13}C-NMR

Analytical data for 4-(2-(5-(((2-(2-(2-aminoethoxy)ethoxy)ethyl)(ethyl)amino)methyl)-2-methoxyphenyl)-1*H*-pyrrolo[2,3-*b*]pyridin-4-yl)-2-isopropylbenzoic acid (**15b**):

^{1}H NMR (500 MHz, MeOD-d_4) δ) 8.24 (1H, d, *J* = 5.1 Hz, Ar*H*), 7.88 (1H, d, *J* = 2.1 Hz, Ar*H*), 7.74 (1H, d, *J* = 1.8 Hz, Ar*H*), 7.58 (1H, dd, *J* = 7.8, 1.8 Hz, Ar*H*), 7.51 (1H, d, *J* = 7.8 Hz, Ar*H*), 7.28 (1H, dd, *J* = 8.4, 2.1 Hz, Ar*H*), 7.21 (1H, d, *J* = 5.1 Hz, Ar*H*), 7.12 (1H, d, *J* = 8.4 Hz, Ar*H*), 7.06 (1H, s, Ar*H*), 4.02 (3H, s, OCH_3), 3.68 (2H, s, ArCH_2N), 3.67–3.53 (7H, m, 3×CH_2, isopropyl C*H*), 3.53–3.49 (2H, m, OCH_2), 2.98–2.89 (2H, m, OCH_2), 2.74–2.68 (2H, m, OCH_2), 2.65 (2H, q, *J* = 7.1 Hz, ethyl NCH_2), 1.37 (6H, d, *J* = 6.8, isopropyl CH_3), 1.11 (3H, t, *J* = 7.1 Hz, ethyl CH_3); ^{13}C NMR (126 MHz, MeOD-d_4) δ 177.3 (*C*), 155.8 (*C*), 148.7 (*C*), 145.3 (*C*), 142.2 (*C*H), 142.0 (*C*), 141.3 (*C*), 138.0 (*C*), 136.8 (*C*), 131.3 (*C*), 130.2 (*C*H), 128.8 (*C*H), 126.7 (*C*H), 125.3 (*C*H), 125.1 (*C*H), 119.6 (*C*), 119.1 (*C*), 114.4 (*C*H), 111.4 (*C*H), 98.0 (*C*H), 69.9 (2xCH_2), 68.9 (CH_2), 66.9 (CH_2), 57.2 (CH_2), 55.0 (CH_3), 52.1 (CH_2), 47.8 (CH_2), 39.3 (CH_2), 30.0 (*C*H), 23.3 (CH_3), 10.4 (CH_3); m/z (ES^+) 575 ($C_{33}H_{43}N_4O_5$, 100%, $[M+H]^+$); HRMS $[C_{33}H_{42}N_4O_5+H]^+$ calcd. 575.3233, found 575.3251 (3.1 ppm).

4-(2-(5-((Diethylamino)methyl)-2-methoxyphenyl)-1-tosyl-1*H*-pyrrolo[2,3-*b*]pyridin-4-yl)-2-isopropylbenzoic acid (16)

To a mixture of *N*-(3-(4-bromo-1-tosyl-1*H*-pyrrolo[2,3-*b*]pyridin-2-yl)-4-methoxybenzyl)-*N*-ethylethanamine (**13b**) (451 mg, 0.83 mmol), 2-isopropyl-4-(4,4,5,5-tetramethyl-1,3,2-dioxaborolan-2-yl)benzoic acid (**11**) (597 mg, 2.06 mmol) in 1,4-dioxane (7.0 mL) was added Pd(dppf)Cl_2 (68 mg, 0.083 mmol) and Na_2CO_3 (264 mg, 2.49 mmol) in water (1.53 mL). The mixture was degassed with N_2 thrice and then stirred at 100°C for 16 h. The reaction mixture was concentrated under reduced pressure to give a residue which was purified using reversed phase prep HPLC (C_{18} silica column, [water (0.1% aq. NH_3) - MeCN] B%: (0 → 100%) to give 4-(2-(5-((diethylamino)methyl)-2-methoxyphenyl)-1-tosyl-1*H*-pyrrolo[2,3-*b*]pyridin-4-yl)-2-isopropylbenzoic acid (**16**) (220 mg, 0.35 mmol, 42%) as a white solid; ^{1}H NMR (400 MHz, MeOD-*d*$_4$) δ 8.33 (1H, d, *J* = 5.1 Hz, Ar*H*), 7.81–7.72 (2H, m, AA′BB′, 2×Ar*H*), 7.65 (1H, s, Ar*H*), 7.60 (1H, d, *J* = 8.3 Hz, Ar*H*), 7.46 (1H, s, Ar*H*), 7.35–7.22 (4H, m, AA′BB′, 4×Ar*H*), 7.21–7.12 (2H, m, 2×Ar*H*), 6.68 (1H, s, Ar*H*), 4.33 (2H, s, ArCH_2N), 3.83 (3H, s, OCH_3), 3.61–3.51 (1H, m, isopropyl C*H*), 3.22 (4H, q, *J* = 7.1 Hz, 2×ethyl CH_2), 2.33 (3H, s, tosyl CH_3), 1.35 (6H, t, *J* = 7.1 Hz, 2×ethyl CH_3), 1.21 (6H, br s, isopropyl CH_3); ^{13}C NMR (101 MHz, MeOD-*d*$_4$) δ 176.5 (*C*), 159.3 (*C*), 149.6 (*C*), 145.7 (*C*), 145.3 (*C*), 144.3 (*C*H), 143.2 (*C*), 141.1 (*C*), 137.6 (*C*), 136.5 (*C*), 135.8 (*C*), 133.5 (*C*H), 133.2 (*C*H), 129.1 (*C*H), 127.4 (*C*H), 127.0 (*C*H), 125.1 (*C*H), 125.0 (*C*H), 122.9 (*C*), 121.6 (*C*), 120.0 (*C*), 118.1 (*C*H), 111.0 (*C*H), 108.0 (*C*H), 55.3 (CH_2), 54.9 (CH_3), 46.4 (CH_2), 29.9 (*C*H), 23.0 (CH_3), 20.1 (CH_3), 7.8 (CH_3); m/z (ES$^+$) 626 ($C_{36}H_{39}N_3O_5S$+H, 100%, $[M+H]^+$).

***Tert*-butyl (2-(2-(2-(4-(2-(5-((diethylamino)methyl)-2-methoxyphenyl)-1-tosyl-1*H*-pyrrolo[2,3-*b*]pyridin-4-yl)-2-isopropylbenzamido)ethoxy)ethoxy)ethyl)carbamate (17)**

To a solution of 4-(2-(5-((diethylamino)methyl)-2-methoxyphenyl)-1-tosyl-1*H*-pyrrolo[2,3-*b*]pyridin-4-yl)-2-isopropylbenzoic acid (**16**) (150 mg, 0.24 mmol) in DMF (7 mL) was added neat DIPEA (98 µL, 0.72 mmol), *tert*-butyl (2-(2-(2-aminoethoxy)ethoxy)ethyl)carbamate (89.3 mg, 0.36 mmol) and HCTU (109 mg, 0.26 mmol). The resultant mixture was then stirred at rt for 16 h. The reaction mixture was filtered and directly purified by reversed phase prep HPLC (C_{18} silica column; mobile phase: 5:95 → 100:0 MeCN:0.1% NH_3 (aq)) to give *tert*-butyl (2-(2-(2-(4-(2-(5-((diethylamino)methyl)-2-methoxyphenyl)-1-tosyl-1*H*-pyrrolo[2,3-*b*]pyridin-4-yl)-2-isopropylbenzamido)ethoxy)ethoxy)ethyl)carbamate (**17**) (200 mg, 0.24 mmol, 97%) as a colourless oil; ^{1}H NMR (400 MHz, MeOD-*d*$_4$) δ 8.45 (1H, d, *J* = 5.0 Hz, Ar*H*), 7.89–7.81 (2H, m, AA′BB′, 2xAr*H*), 7.71 (1H, s, Ar*H*), 7.58 (1H, dd, *J* = 7.8, 1.3 Hz, Ar*H*), 7.54–7.47 (2H, m, 2xAr*H*), 7.45–7.38 (2H, m, 2xAr*H*), 7.39–7.31 (2H, m, AA′BB′, 2xAr*H*), 7.10 (1H, d, *J* = 8.3 Hz, Ar*H*), 6.70 (1H, s, Ar*H*), 3.86 (3H, s, OCH_3), 3.79–3.61 (9H, m, 4xCH_2, isopropyl C*H*), 3.61–3.55 (2H, m, CH_2), 3.40 (2H, s, ArCH_2N), 3.30–3.23 (2H, m, CH_2), 2.69 (4H, q, *J* = 7.1 Hz, 2×ethyl CH_2), 2.42 (3H, s, tosyl CH_3), 1.45 (9H, s, ^{t}Bu-*Me*$_3$), 1.37-1.36 (6H, m, isopropyl CH_3), 1.18 (6H, t, *J* = 7.1 Hz, 2×ethyl CH_3); ^{13}C NMR (101 MHz, MeOD-*d*$_4$) δ 171.6 (*C*), 157.6 (*C*), 149.6 (*C*), 147.0 (*C*), 145.2 (*C*), 144.0 (*C*H), 142.0 (*C*), 139.3 (*C*), 138.8 (*C*), 136.8 (*C*), 136.1 (*C*), 132.1 (*C*H), 131.7 (*C*H), 129.5 (*C*), 129.0 (*C*H), 127.5 (*C*H), 127.3 (*C*H), 125.7 (*C*H), 125.6 (*C*H), 121.6 (*C*), 120.1 (*C*), 118.1 (*C*H), 110.1 (*C*H), 107.0 (*C*H), 78.7 (*C*), 69.9 (CH_2), 69.7 (CH_2), 69.1 (CH_2), 56.1 (CH_2), 54.7 (CH_3), 46.0 (CH_2), 39.8 (CH_2), 39.3 (CH_2), 29.9 (*C*H), 29.3 (CH_2), 27.4 (CH_3), 23.1 (CH_3), 20.1 (CH_3), 10.1 (CH_3); m/z (ES$^+$) 856 ($C_{47}H_{61}N_5O_8S$, 100%, $[M]^+$).

Note: one of the aromatic quaternary carbons was not visible in the ^{13}C-NMR

***Tert*-butyl (2-(2-(2-(4-(2-(5-((diethylamino)methyl)-2-methoxyphenyl)-1*H*-pyrrolo[2,3-*b*]pyridin-4-yl)-2-isopropylbenzamido)ethoxy)ethoxy)ethyl)carbamate (18a)**

To a suspension of *tert*-butyl (2-(2-(2-(4-(2-(5-((diethylamino)methyl)-2-methoxyphenyl)-1-tosyl-1*H*-pyrrolo[2,3-*b*]pyridin-4-yl)-2-isopropylbenzamido)ethoxy)ethoxy)ethyl)carbamate (**17**) (243 mg, 0.284 mmol) and K_2CO_3 (79 mg, 0.568 mmol) was dissolved in MeOH (1.80 mL) and the reaction stirred at 65°C for 16 h. The reaction mixture was then concentrated under reduced pressure and purified by reverse phase prep HPLC (C_{18} column; mobile phase: 5:95 → 100:0 MeCN:0.1% NH_3 (aq)) to give *tert*-butyl (2-(2-(2-(4-(2-(5-((diethylamino)methyl)-2-methoxyphenyl)-1*H*-pyrrolo[2,3-*b*]pyridin-4-yl)-2-isopropylbenzamido)ethoxy)ethoxy)ethyl)carbamate (**18a**) (90 mg, 0.12 mmol, 43%) as a colourless oil; ^{1}H NMR (500 MHz, MeOD-d_4) δ 8.24 (1H, d, *J* = 5.1 Hz, Ar*H*), 7.83 (1H, d, *J* = 1.8 Hz, Ar*H*), 7.78 (1H, d, *J* = 2.1 Hz, Ar*H*), 7.65 (1H, dd, *J* = 7.8, 1.8 Hz, Ar*H*), 7.48 (1H, d, *J* = 7.8 Hz, Ar*H*), 7.30 (1H, dd, *J* = 8.4, 2.1 Hz, Ar*H*), 7.21 (1H, d, *J* = 5.1 Hz, Ar*H*), 7.11 (1H, d, *J* = 8.4 Hz, Ar*H*), 7.08 (1H, s, Ar*H*), 3.98 (3H, s, OCH_3), 3.71–3.59 (10 H, m, ArCH_2N, 4×CH_2), 3.54–3.50 (2H, m, OCH_2), 3.42–3.36 (1H, m, isopropyl C*H*), 3.34 (1H, s, N*H*), 3.23–3.19 (2H, m, CH_2O), 2.56 (4H, q, *J* = 7.2 Hz, 2×ethyl CH_2), 1.38 (9H, s, ^{t}Bu-*Me*$_3$), 1.36 (6H, d, *J* = 6.9 Hz, isopropyl CH_3), 1.08 (6H, t, *J* = 7.2 Hz, 2×ethyl CH_3); ^{13}C NMR (126 MHz, MeOD-d_4) δ 171.9 (*C*), 155.9 (*C*), 148.8 (*C*), 146.7 (*C*), 142.2 (*C*H), 141.1 (*C*), 140.2 (*C*), 136.8 (*C*), 136.3 (*C*), 130.7 (*C*), 130.5 (*C*H), 129.0 (*C*H), 127.2 (*C*H), 125.8 (*C*H), 125.5 (*C*H), 119.5 (*C*), 119.2 (*C*), 114.5 (*C*H), 111.4 (*C*H), 98.5 (*C*H), 78.7 (*C*), 70.0 (*C*H_2), 69.9 (*C*H_2), 69.7 (*C*H_2), 69.2 (*C*H_2), 56.2 (*C*H_2), 54.9 (*C*H_3), 46.0 (*C*H_2), 39.8 (*C*H_2), 39.4 (*C*H_2), 30.0 (*C*H), 27.3 (*C*H_3), 23.2 (*C*H_3), 10.0 (*C*H_3); m/z (ES^+) 702 ($C_{40}H_{56}N_5O_6$, 100%, $[M+H]^+$); HRMS $[C_{40}H_{55}N_5O_6+H]^+$ calcd. 702.4231, found 702.4264 (4.7 ppm).

Note: one of the aromatic quaternary carbons was not visible in the ^{13}C-NMR

***N*-(2-(2-(2-aminoethoxy)ethoxy)ethyl)-4-(2-(5-((diethylamino)methyl)-2-methoxyphenyl)-1*H*-pyrrolo[2,3-*b*]pyridin-4-yl)-2-isopropylbenzamide (18b)**

To *tert*-butyl (2-(2-(2-(4-(2-(5-((diethylamino)methyl)-2-methoxyphenyl)-1*H*-pyrrolo[2,3-*b*]pyridin-4-yl)-2-isopropylbenzamido)ethoxy)ethoxy)ethyl)carbamate (**18a**) (17 mg, 0.024 mmol) was added an ice-cold solution of 1:2 TFA/CH_2Cl_2 (3 mL). The reaction mixture was allowed to warm to rt and stirred for a further 2 h. The reaction mixture was subsequently concentrated under reduced pressure and loaded onto an SCX cartridge in a minimum volume of MeOH. The SCX cartridge was washed with 3 column volumes of MeOH, followed by 3 column volumes of 7 N ammonia in MeOH to elute the title compound as a yellow oil, (16 mg, 0.024 mmol, 99%); ^{1}H NMR (500 MHz, MeOD-d_4) δ 8.24 (1H, d, *J* = 5.1 Hz, Ar*H*), 7.83 (1H, d, *J* = 1.7 Hz, Ar*H*), 7.77 (1H, d, *J* = 1.9 Hz, Ar*H*), 7.64 (1H, dd, *J* = 7.8, 1.7 Hz, Ar*H*), 7.46 (1H, d, *J* = 7.8 Hz, Ar*H*), 7.29 (1H, dd, *J* = 8.4, 1.9 Hz, Ar*H*), 7.20 (1H, d, *J* = 5.1 Hz, Ar*H*), 7.12–7.04 (2H, m, 2×Ar*H*), 3.97 (3H, s, OCH_3), 3.70–3.57 (10H, m, ArCH_2N, 4×CH_2), 3.58–3.54 (2H, m, CH_2), 3.42–3.35 (1H, m, isopropyl C*H*), 2.86–2.76 (2H, m, CH_2), 2.56 (4H, q, *J* = 7.1 Hz, 2×ethyl CH_2), 1.35 (6H, d, *J* = 6.9 Hz, isopropyl CH_3), 1.08 (6H, t, *J* = 7.2 Hz, 2×ethyl CH_3); ^{13}C NMR (126 MHz, MeOD-d_4) δ 171.9 (*C*), 156.0 (*C*), 148.9 (*C*), 146.7 (*C*), 142.2 (*C*H), 141.1 (*C*), 140.2 (*C*), 136.8 (*C*), 136.3 (*C*), 130.6 (*C*H), 129.1 (*C*H), 127.2 (*C*H), 125.8 (*C*H), 125.5 (*C*H), 119.5 (*C*), 119.2 (*C*), 114.5 (*C*H), 111.4 (*C*H), 98.5 (*C*H), 71.3 (*C*H_2), 70.0 (*C*H_2), 69.9 (*C*H_2), 69.2 (*C*H_2), 56.2 (*C*H_2), 54.9 (*C*H_3), 46.0 (*C*H_2), 40.5 (*C*H_2), 39.3 (*C*H_2), 30.0 (*C*H), 23.2 (*C*H_3), 10.0 (*C*H_3); m/z (ES^+) 602 ($C_{35}H_{47}N_5O_4$, 40%, $[M+H]^+$); HRMS $[C_{35}H_{47}N_5O_4+H]^+$ calcd. 602.3706, found 602.3729 (3.8 ppm).

Supplemental references

1. Guntur, G.; Gleeson, D.; Anderson, M.; Mutter, N.; Webster, L.; Read, K. D.; Gleeson, M. P., Computationally guided optimization of the antimalarial activity and physicochemical properties of 2,4-diaminopyrimidines. *RSC Med Chem* **2025**.
2. Santosh Kumar, S. C.; Vijendra Kumar, N.; Srinivas, P.; Bettadaiah, B. K., A Convenient Practical Synthesis of Alkyl and Aryl Oxime Esters. *Synthesis* **2014,** *46* (14), 1847–1852.
3. Mahindra, A.; Janha, O.; Mapesa, K.; Sanchez-Azqueta, A.; Alam, M. M.; Amambua-Ngwa, A.; Nwakanma, D. C.; Tobin, A. B.; Jamieson, A. G., Development of Potent PfCLK3 Inhibitors Based on TCMDC-135051 as a New Class of Antimalarials. *J Med Chem* **2020,** *63* (17), 9300–9315.
